# Selective thiazoline peptide cyclisation compatible with mRNA display and efficient synthesis†

**DOI:** 10.1039/d3sc03117a

**Published:** 2023-09-08

**Authors:** Minglong Liu, Richard Morewood, Ryoji Yoshisada, Mirte N. Pascha, Antonius J. P. Hopstaken, Eliza Tarcoveanu, David A. Poole, Cornelis A. M. de Haan, Christoph Nitsche, Seino A. K. Jongkees

**Affiliations:** a Department of Chemistry and Pharmaceutical Sciences, Vrije Universiteit Amsterdam Amsterdam The Netherlands S.A.K.Jongkees@vu.nl; b Amsterdam Institute of Molecular and Life Sciences (AIMMS), Vrije Universiteit Amsterdam Amsterdam The Netherlands; c Research School of Chemistry, Australian National University Canberra ACT 2601 Australia christoph.nitsche@anu.edu.au; d Section Virology, Division Infectious Diseases and Immunology, Department of Biomolecular Health Sciences, Faculty of Veterinary Medicine, Utrecht University Yalelaan 1 3584 CL Utrecht The Netherlands

## Abstract

Peptide display technologies are a powerful method for discovery of new bioactive sequences, but linear sequences are often very unstable in a biological setting. Macrocyclisation of such peptides is beneficial for target affinity, selectivity, stability, and cell permeability. However, macrocyclisation of a linear hit is unreliable and requires extensive structural knowledge. Genetically encoding macrocyclisation during the discovery process is a better approach, and so there is a need for diverse cyclisation options that can be deployed in the context of peptide display techniques such as mRNA display. In this work we show that *meta*-cyanopyridylalanine (*m*CNP) can be ribosomally incorporated into peptides, forming a macrocycle in a spontaneous and selective reaction with an N-terminal cysteine generated from bypassing the initiation codon in translation. This reactive amino acid can also be easily incorporated into peptides during standard Fmoc solid phase peptide synthesis, which can otherwise be a bottleneck in transferring from peptide discovery to peptide testing and application. We demonstrate the potential of this new method by discovery of macrocyclic peptides targeting influenza haemagglutinin, and molecular dynamics simulation indicates the *m*CNP cross-link stabilises a beta sheet structure in a representative of the most abundant cluster of active hits. Cyclisation by *m*CNP is also shown to be compatible with thioether macrocyclisation at a second cysteine to form bicycles of different architectures, provided that cysteine placement reinforces selectivity, with this bicyclisation happening spontaneously and in a controlled manner during peptide translation. Our new approach generates macrocycles with a more rigid cross-link and with better control of regiochemistry when additional cysteines are present, opening these up for further exploitation in chemical modification of *in vitro* translated peptides, and so is a valuable addition to the peptide discovery toolbox.

## Abstract 2

A new tool for peptide discovery. Pyridine-nitrile based macrocyclisation in mRNA display format, provides novel conformational constraints and allows construction of controlled bicyclic structures.

## Graphical abstract

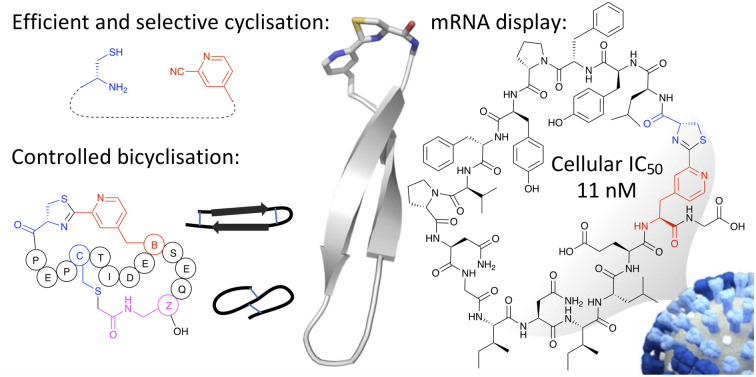

## Introduction

Peptides are currently experiencing a surge of interest as drug candidates,^1^ arising from their strong target engagement, selective binding, and low immunogenicity.^2^ However, they still have major drawbacks in their pharmacokinetics, as they are often degraded quickly. Modified peptides, or peptides with non-natural building blocks, can offer solutions to these challenges, and prime among these is the macrocyclisation of the peptide.^3^ This removes one or both termini from access to proteases, while conformational constraint can further reduce access to endopeptidases. It also removes some of the entropic penalty to binding by holding the peptide in a (near-)active conformation, and can improve selectivity by preventing alternate conformations. Macrocyclisation is likely the most impactful single change that can be made to a peptide to address many of the problems associated with their use in a biological setting, although it is rarely enough on its own.^4,5^

The approach used for peptide macrocyclisation can have a large influence on biological activity.^6,7^ Macrocyclisation is typically well tolerated if it is already present at the discovery stage of ‘*de novo*’ peptides (those derived from random peptide libraries^8^), as the peptide will be selected to fit the conformational constraints imposed by the cyclisation approach. This means that changes during lead development are minimised. By contrast, macrocyclisation introduced at a later stage can be much more difficult to optimise, requiring extensive structural information and trial and error.^9,10^ There is thus a demand for peptide macrocyclisation approaches that are compatible with both ribosomal translation and with peptide production techniques such as solid-phase peptide synthesis (SPPS) or bacterial expression.

The peptide discovery technique of mRNA display is particularly suited to discovery of new macrocyclic peptides as the peptide libraries are generated by *in vitro* translation.^11^ In this, the building block pool can be easily manipulated, both by addition of new building blocks and by omission of canonical amino acids to generate vacant codons, resulting in a shift in the chemical space of the displayed peptides. A particularly convenient method for adding new building blocks is through the use of aminoacylating ribozymes called flexizymes.^12^ These recognise only the CCA acceptor stem of tRNA, so are anticodon-agnostic. They are also promiscuous in substrate scope, having been evolved to recognise only an aromatic group in either the amino acid or activated ester. This combination of flexible *in vitro* translation by means of flexizyme with peptide enrichment against a biologically relevant target using mRNA display has been termed the random non-standard peptide integrated discovery (RaPID) system.^13^

**Fig. 1:**
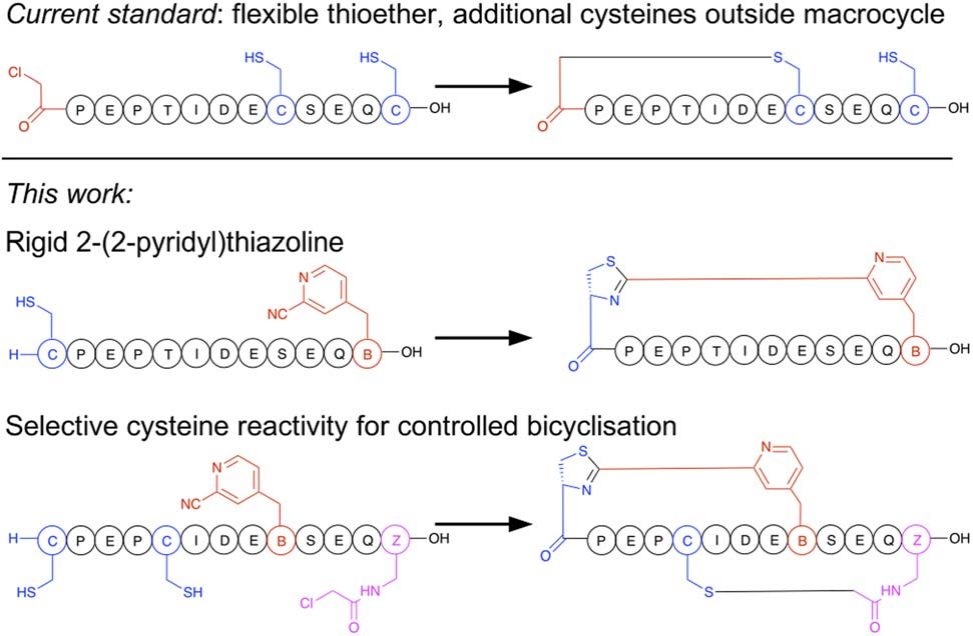
Comparison of selectivity between the established approach to peptide macrocyclisation in mRNA display with that in the current work.

Macrocyclisation in the RaPID system is at present carried out predominantly by initiating peptide translation with a chloroacetylated aromatic amino acid, although a few other methods have been developed.^14^ This chloroacetyl moiety reacts with the first downstream cysteine to give a stable non-reducible thioether linkage (Fig. 1).^15^ This has many favourable properties, including spontaneous and fast reaction, predictable ring size, and easy scalability in SPPS. However, with the exception of the second position, it cannot produce macrocycles that contain additional cysteine residues (which are particularly useful as reactive handles for further chemical or enzymatic modification^16,17^ and for access to multicyclic peptides), it generates a flexible linkage, and it cannot currently be accessed during bacterial expression. Therefore, an increased diversity in macrocyclisation approaches is necessary, which will also give rise to a corresponding increase in diversity of peptide structures and as a result may further increase the scope of targets accessible to this method. A macrocyclisation approach that works well for one target may not work well for another, and so it is desirable to have a panel of different methods that together increase the chance of success.

The N-terminus of a peptide or protein is unique in its reactivity,^18,19^ having a different p*K*_a_ and having neighbouring functional groups not present in lysine, which is the other common amine in peptides and proteins. A host of methods have been developed for specific modification of the N-terminus of a protein, and these can thus serve as inspiration for functional groups that can be incorporated into amino acids for translation into peptides, and thereby afford new and more selective cyclisation reagents. We have recently reported a facile non-enzymatic approach to access peptides in bacterial *in vitro* translation that does not contain a formylated methionine, and so has a free N-terminus available for reaction.^20^ Of the N-terminal selective functional groups, aromatic electrophilic nitriles have recently caught broad attention for their rapid formation of a thiazoline heterocycle with an N-terminal cysteine. This reaction can be efficiently exploited in peptide macrocyclisation by incorporation of *meta*-cyanopyridylalanine (*m*CNP) at the C-terminus of a peptide.^21^ Based on this, we also recently reported a method for translation of a cyanobenzothiazole-containing amino acid that gave efficient peptide macrocyclisation,^22^ while others have exploited this same reactive group together with a second cysteine-reactive moiety for stapling of peptides displayed on phage,^23,24^ with selectivity arising from either differences in rate or reversibility of the second reaction. However, the high reactivity of cyanobenzothiazoles meant that extra precautions needed to be taken during translation of this moiety to prevent premature hydration. A cyanobenzothiazole-containing amino acid was also not directly amenable to SPPS, and so needed to be incorporated in its thiazoline form with the cysteine carboxylate orthogonally protected to allow a late-stage amide bond-forming macrocyclisation. This low yielding and labour-intensive scale-up is thus a hurdle to application.

In the current work we present a facile approach to peptide macrocyclisation in mRNA display (Fig. 1), overcoming all hurdles from our previous studies. By modifying the reactivity of the aromatic nitrile, in the form of a ribosomally translatable *meta*-pyridine nitrile amino acid, all side reactions can be prevented and SPPS becomes routine, while still retaining sufficiently fast macrocyclisation for application in library building in the RaPID system. Notably this new approach has selectivity over additional cysteine residues, which we show can be exploited in controlled bicyclisation during *in vitro* translation.

## Results and discussion

### Flexible *in vitro* translation of a pyridine nitrile amino acid

**Fig. 2:**
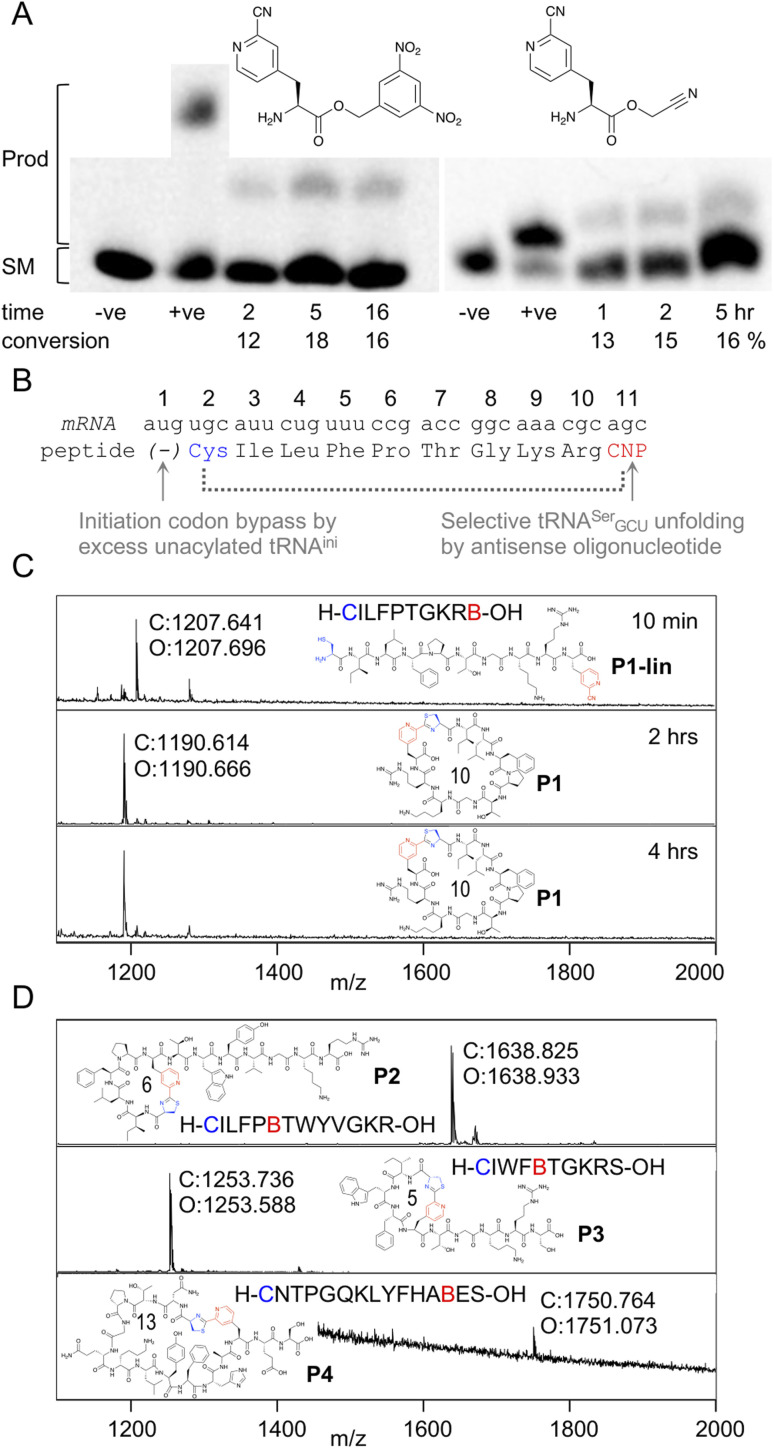
Aminoacylation and translation with *m*CNP. (A) Charging of activated esters shown onto a fluorescein-labeled 5-base tRNA analogue, analysed by urea PAGE. Starting material RNA and product acylated RNA are indicated to the left and time points below the gel, along with negative control ‘−ve’ (DMSO only) and positive control ‘+ve’ (Lys-DBE for 5 hours and Ac-Phe-CME for 2 hours) for each. Conversions are estimated by densitometry of fluorescent scan of gel. Full gels are presented in Fig. S1 and S2.† (B) Schematic representation of the template sequence used to test *m*CNP incorporation by *in vitro* translation, indicating cyclisation by dotted bracket and annotating how each relevant codon was liberated. CNP = *meta*-cyanopyridylalanine (C) MALDI-TOF-MS spectra after varying time points for peptide translations using tRNA acylated with *m*CNP by dFx, showing calculated (*C*) and observed (*O*) masses. Peptide linear sequence and structure is as indicated with ‘B’ as one-letter code for *m*CNP, with ring size in amino acids also shown. Pyridine nitrile (derived) moieties are indicated in red and 1,2-aminothiol (derived) moieties in blue. Peptide calculated and observed masses are shown by the peak. (D) MALDI-TOF-MS spectra for peptide translations with varied ring sizes and amino acid compositions, coloured as for B.

In order to be able to charge *m*CNP onto tRNA by flexizyme, two activated esters were synthesised with cyanomethyl ester and dinitrobenzyl ester leaving groups. These esters serve as substrates for eFx and dFx type flexizymes, respectively, with eFx and cyanomethyl esters typically being used for aromatic amino acids. Both options were synthesised here to increase the chance of success. These were prepared by alkylation of the Boc-protected amino acid with the relevant alkyl chloride before deprotection under acidic conditions. Using these activated esters, acylation onto a fluorescent 5-base tRNA mimetic substrate was assessed by urea PAGE analysis after varied incubation times (Fig. 2A).^25^ These showed acylation occurring for both substrates, with the reaction peaking after 5 hours incubation at 18% for dFx and at 16% for eFx. However, we observed hydrolytic instability during storage with the cyanomethyl ester, and so we opted to continue with the more stable dinitrobenzyl ester for further experiments despite this being atypical for aromatic amino acids.

Translation was tested in a model peptide with sequence H-CILFPTGKRB-OH (P1, where B is used as a one letter code for *m*CNP). Methionine was omitted from the translation, and an excess of non-acylated initiator tRNA was used to increase the efficiency of translation start from the second codon (cysteine). The *m*CNP amino acid was charged onto the engineered^26^ tRNA^EnAsnE2^_GCU_ to reprogram the serine AGC codon and native tRNA^Ser^_GCU_ was sequestered by binding to an antisense oligonucleotide (Fig. 2B).^27^ Observing the product by MALDI-TOF-MS after 10 min of translation showed a peak matching the expected mass for the linear product (Fig. 2C), while after 30 min translation and a further 1.5 hour incubation only the macrocyclic product was observed. A longer incubation, for a total of 4 hours, showed no further change to this product and thus confirms that the macrocyclic peptide is stable in the *in vitro* translation reaction mixture across the range of time spans typically involved in mRNA display.

We next assessed the ability of the macrocyclisation to tolerate different ring sizes and amino acid compositions in peptides P2 through P4 (Fig. 2D). Rings of 5, 6, 10, and 13 amino acids were all well tolerated (including N-terminal Cys and *m*CNP in the ring size), showing only macrocyclic product after 30 min of translation and 1.5 hours of cyclisation. Collectively, these sequences cover the canonical amino acids most likely to interfere with the reaction (S, T, Y, R, K, H), and no signs were seen of side-reactions. These peptides span the size range typically used for mRNA display experiments using the RaPID system, and so indicate that the *m*CNP cyclisation is well suited to this application.

**Fig. 3:**
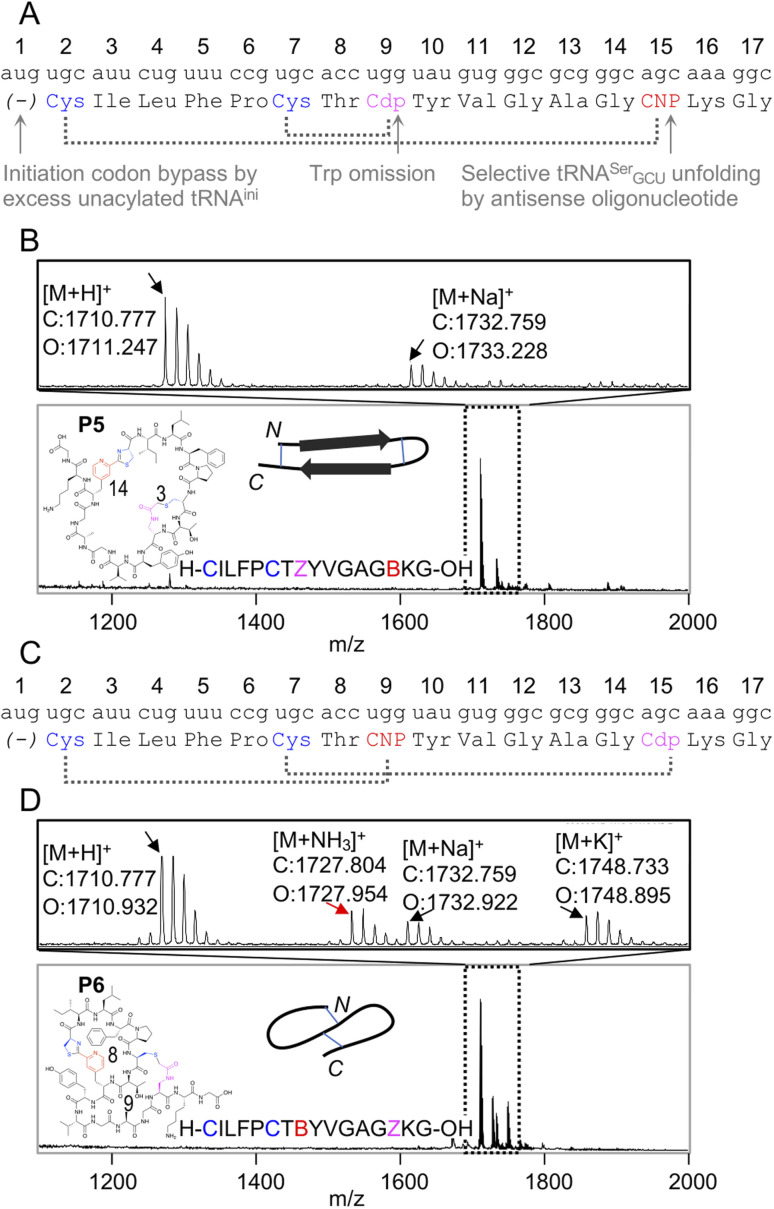
Controlled bicylisation of a test peptide with two different cysteine-reactive electrophiles; chloroacetamide and pyridine nitrile. (A) Schematic representation of the test template and reprogrammed translation, as for Fig. 2B. Cdp = l-*N*-β-chloroacetyl 2,3-diaminopropionic acid. (B) MALDI-TOF-MS showing peptide product matching calculated mass for bicyclisation, with expansion above showing specific peaks and their identities. Overlaid on the spectrum is the product structure, a cartoon representation of cyclisation pattern, and the linear sequence of the peptide (with ‘B’ as one-letter code for *m*CNP and ‘Z’ for ClAc-Dap, and ring sizes in amino acids indicated). (C) As for (A), but with alternate codon assignments for the two reactive amino acids. (D) As for (B), but showing a minor product with ammonia adduct consistent with incorrect macrocyclisation (red arrow).

Because the *m*CNP cyclisation is selective for the 1,2-aminothiols of N-terminal cysteines,^21^ it was anticipated to be tolerant of other cysteines in the peptide. To demonstrate the utility of this we translated a further two test peptides P5 and P6. These each contained two cysteines, one at the start and another in the middle of the sequence (Fig. 3). In these we also translated two reactive groups, *m*CNP and l-*N*-β-chloroacetyl 2,3-diaminopropionic acid (ClAc-Dap or Cdp). This second non-canonical amino acid was incorporated by acylation onto tRNA^EnAsnE2^_CCA_ by dFx from a dinitrobenzyl ester^28^ and translated at a vacant codon created by further omission of tryptophan in addition to methionine from the translation reaction solution. P5 and P6 differ in the relative placements of these reactive groups, to access different peptide crosslinking architectures.

In designing these test peptides, we considered two selectivity ‘rules’ for the two reactive groups: chloroacetamides have previously been shown to have good selectivity for the closest thiol when incorporated at the N-terminus.^15^ and *m*CNP has been shown to form stable products exclusively with an N-terminal cysteine.^21^ Of these, the chloroacetamide is the less selective, and so imposes constraints on the design. As long as the ClAc-Dap is placed so that the closest cysteine is not the N-terminal cysteine, we expect both selectivities to reinforce one another and bicyclisation to proceed with sufficient control. Thus, while these two reactive groups are not orthogonal, careful peptide design can give spontaneous and controlled bicyclisation.

Important for characterisation of the product is that both cyclisations are able to be independently monitored in MALDI-TOF-MS through the loss of masses corresponding to the Cl and NH_3_ leaving groups (from ClAc-Dap and *m*CNP, respectively). Undesired reaction of the N-terminal cysteine with the internal ClAc-Dap would then be expected to retain the NH_3_ leaving group, with the internal cysteine unable to form a stable adduct to *m*CNP.

We observed only the mass corresponding to the bicyclic product for P5, demonstrating the formation of a large macrocycle with a further staple as could be used in stabilisation of a beta sheet structure.^29^ In P6, these reactive groups were reversed in an otherwise identical background to instead give a ‘figure 8’ type structure. Notably, this spacing of *i*; *i* + 7 or *i* + 8 could be expected to stabilise a helical fold, and again for P6 the major product was the correctly bicyclised peptide. In this second case a minor peak was also observed that corresponds to the mass of an ammonia adduct, and thus indicates alternative cyclisation, but such a minor side product is not anticipated to be a problem in applying this method for bicyclic library construction.

These two peptides thus demonstrate that the two cysteine-based reactions can be used together for controlled peptide bicyclisation following *in vitro* translation despite not being fully orthogonal. Placement of the chloroacetamide C-terminal to both cysteines was expected to maximise selectivity of this less-selective pair, allowing it to exploit differences in reactivity from cysteine proximity. We observed that in P5 the selectivity was better, and so this indicates that selectivity of this reaction benefits from being in a smaller macrocycle (3 amino acids in P5*vs.* 9 in P6). While placement of the reactive groups is constrained by these factors, and so this method cannot access all possible bicyclisation architectures, we see no reason all other positions could not be randomised to generate bicyclic peptide libraries in future.

In the setting of reprogrammed *in vitro* translation, the *m*CNP macrocyclisation reaction thus proved to be robust, selective, and efficient, consistent with previous literature on cyclisation following SPPS^21^ and bacterial translation using an orthogonal aaRS enzyme.^30^ Its complementarity to other thiol-based cyclisations also allows spontaneous ribosomal generation of peptides with more complex bicyclic architectures, provided that cysteine placement is carefully considered.

### Selection of thiazoline-macrocyclised peptides targeting influenza haemagglutinin

With this validation of incorporation by *in vitro* translation and subsequent macrocyclisation, we next investigated the application of *m*CNP macrocyclisation in mRNA display selection. For this we took as model target the haemagglutinin (HA) protein from influenza A virus, responsible for entry into host cells through its receptor binding and fusion activity and a potential target for new treatments. It is currently largely targeted by antibodies and hence smaller modalities that capture the same type of interaction are advantageous. We have previously carried out a RaPID selection campaign against this target using thioether macrocyclisation,^31^ and we hypothesised that a new macrocyclisation approach would give rise to a different pool of sequences, potentially also binding at different sites.

A new mRNA display library was designed that contained standard sites for T7 RNA polymerase recognition and ribosome binding, an encoding region for a random peptide of sequence MCX_15_MGGAGAS (NNK codons for X), and finally a puromycin oligonucleotide annealing site to allow mRNA display using the TRAP variant.^32^ For this initial validation of the method, we opted for a relatively simple monocyclic library. Translation of this library was carried out similar to above, with the initiating methionine codon bypassed by addition of an excess of uncharged synthetic initiator tRNA and omission of methionine to give translation start from the second codon (a fixed N-terminal Cys), while the C-terminal methionine codon was recoded to *m*CNP charged onto tRNA^EnAsnE2^_CAU_ by dFx. Additional cysteine and methionine codons can occur within the random regions, but the former was shown above to be compatible with macrocyclisation while the latter would potentially give multiple regioisomers which could in principle be deconvoluted after hit identification if needed and should therefore not be detrimental to library enrichment.

**Fig. 4:**
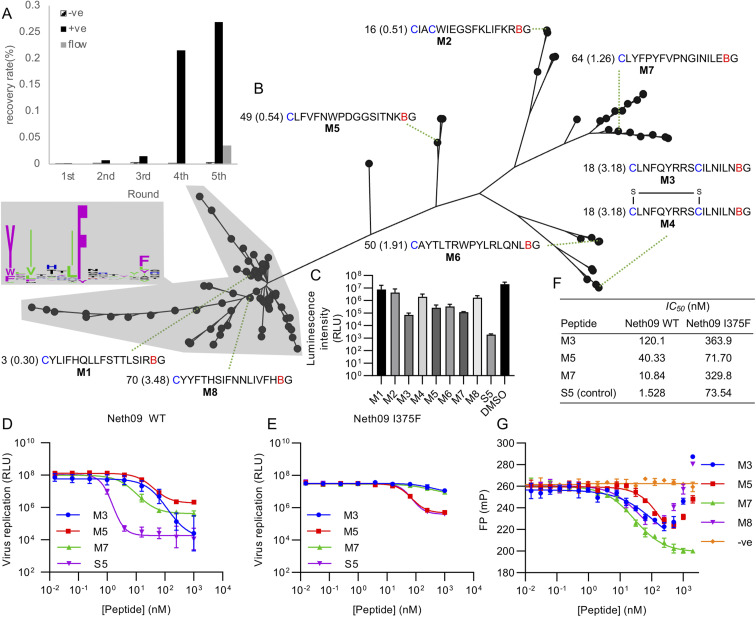
Selection of *m*CNP-macrocyclised peptides targeting influenza haemagglutinin (subtype H1, isolate A/Netherlands/602/2009 – ‘Neth09’). (A) Enrichment of library across selection rounds, plotting recovered DNA as percentage of input library. Positive (‘+ve’) indicates recovery with HA target immobilised on magnetic beads, negative (‘−ve’) indicates direct recovery with the Streptactin-coated magnetic beads used for target immobilisation, and ‘flow’ indicates washing under continuous flow conditions. (B) Phylogenetic tree representation for alignment of high throughput sequencing results from round 5 shown in panel (A), with sequence logo showing conservation by letter height in the main cluster highlighted. Sequences chosen for synthesis are named M1 through M8, with the sequences preceded by the abundance rank as well as the difference in recovery by flow washing *vs.* batch washing in parentheses (high number indicates higher sequence count following flow washing). B indicates *m*CNP, and residues are coloured as in Fig. 1. M4 is a disulfide-cyclised variant of M3. (C) Screen for peptide inhibition activity at 1 μM peptide with H1N1pdm09 virus (A/Netherlands/602/2009) in a luciferase reporter assay. S5 is a positive control thioether-macrocyclised peptide inhibitor from previous work.^31^ (D) Inhibition of infection in the same assay from active peptides derived from this work, along with positive control peptide S5. (E) As for C, but with an I375F resistance mutant. (F) Calculated IC_50_ values from panels (D) and (E). (G) Fluorescence polarisation competition assay with fluorescein-tagged S5 as probe and H1 as target. An unrelated SARS-CoV-2 spike-binding peptide S1b3inL1 was used as negative control (‘−ve’).^34^ Data are shown as mean ± SD for biological replicates (*n* = 2 for panel (C), 3 for panels (D), (E) and (G)).

Using this library, five rounds of selection were carried out with clear enrichment across these (Fig. 4A). The enriched library from the fourth round was then also subjected to a further round using continuous flow rather than batch washing, to attempt to drive further enrichment of sequences with a low off-rate.^33^ This showed a large drop in recovery, but still above background. Negative control recovery with the same Strep-Tactin beads used for target immobilisation remained consistently low. Enriched libraries were then sequenced in high-throughput to identify candidate hits.

Analysis of the sequencing results revealed one main sequence family with a central YXIXXXIF motif (Fig. 4B), as well as several other smaller and less well conserved sequence families. We did not find any sequences with clear homology to hits from our previous work,^31^ suggesting that the alternate cyclisation developed here influences the pool of candidates to adopt different interactions with the target. From these sequence families we selected one representative member each (named M1 through M8), covering sequences that were both more and less strongly enriched in the final continuous-flow round to determine if this late-stage application of flow-based enrichment was helpful in finding more potent hits. We also chose two sequences containing additional cysteine residues (M2 and M3/M4). These were then synthesised on solid phase by standard Fmoc SPPS as previously described,^21^ and purified by HPLC. Unlike our previously reported cyanobenzothiazole-based macrocyclisation, this pyridine nitrile building block is stable to piperidine and does not need additional protection, making it much more convenient in scale-up. For one of the peptides with two cysteines we isolated two forms, one correctly cyclised (M3) and one forming a disulfide and retaining the *m*CNP residue (M4). As this could potentially be a relevant form present in the selection, we decided to test both rather than reduce the disulfide and force macrocyclisation.

All synthesised peptides were tested for inhibition using a cell-based luciferase infection assay with H1 (Fig. 4C). Screening at a single concentration showed clear infection inhibition for M3, M5, M6, and M7, while a similar assay with a mutant (I375F) resistant against our previous hits showed most potent activity with M5 (Fig. S3†). Notably, we found minimal to no activity with either M1 or M8, which were representatives of the main cluster of hits. Further profiling of the most promising hits M3, M5 and M7 showed the most potent hit to be M7 (IC_50_ of 10 nM; Fig. 4D and F), while against the resistance mutant M5 showed essentially unchanged activity (IC_50_ 50 nM WT *vs.* 70 nM I375F, Fig. 4E and F). This activity clearly demonstrates that our test selection was successful. M4, the disulfide variant of M3, did not show inhibition and so indicates that the correctly cyclised form was likely present during the selection and that the disulfide formation is a result of the different conditions in SPPS macrocyclisation *vs.* after *in vitro* translation. We did not observe any pattern in activity correlating with increased recovery in the continuous flow selection round, but we did not test binding kinetics directly and hence it remains possible that this would show a clearer effect in the off-rate.

To further assess the influence of our new cyclisation approach on peptide binding to the target, we used a competitive fluorescence polarisation assay to determine if the new hits identified here are likely to be binding at the same site as our previous hits (Fig. 4G). We also tested one of the non-active peptides from the main sequence family to determine if these might be inactive because of binding in a different location, and further included as a negative control our recently reported SARS-CoV-2 spike protein binding peptide.^34^ Polarisation was seen to decrease for all new hits in a concentration-dependent manner, but eventually increased again for all but M7. We attribute this increase to non-specific aggregation effects at higher concentrations. These results clearly show that peptides M3, M5, and M7 all compete with fluorescein-labelled S5 for binding to H1 protein, and that peptide M8 also showed competition despite not being active. Hydrogen–deuterium exchange footprinting was previously used to show that all of our earlier hits bind to helix A in the stem of HA and thereby prevent conformational changes leading to fusion of viral and endosomal membranes,^31^ and this pattern appears to have been maintained in this new library. That the inactive peptide M8 still shows competition suggests it is binding in a partially overlapping site that does not have the same influence on HA conformational dynamics, and thus does not lead to inhibition. This likely also explains why M5 and M7 plateau at less than full inhibition. It remains unclear why this site is so strongly dominant in our *in vitro* peptide selection experiments when the sialic-acid binding ‘head’ domain is dominant for antibodies,^35^ but the high conservation of this region makes it promising for the development of broadly-active macrocyclic peptides.^36^

### Structural effects of thiazoline macrocyclisation

**Fig. 5:**
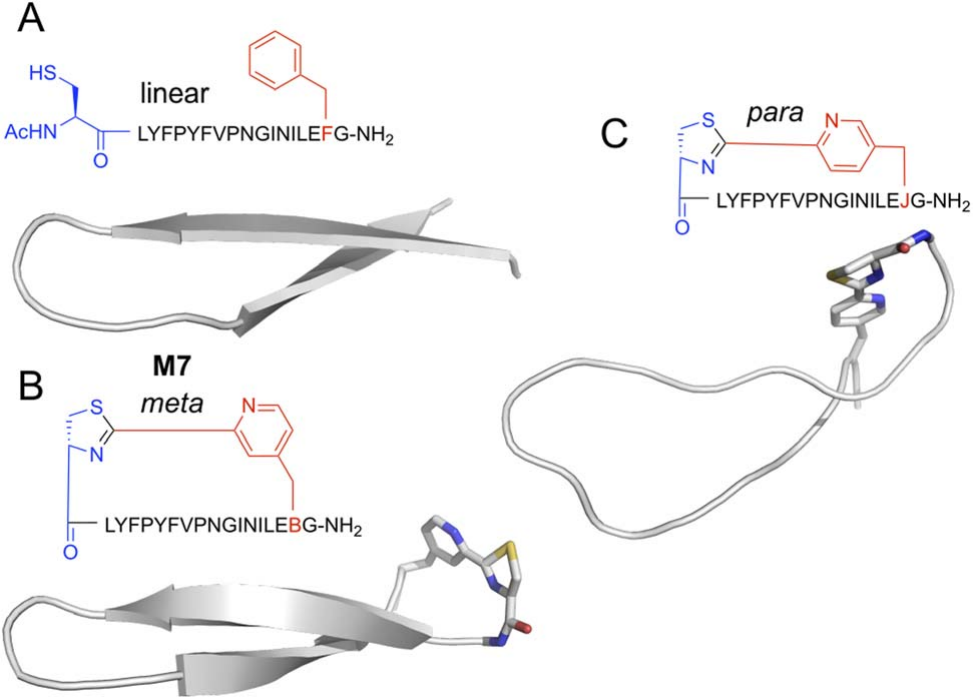
Representative structures for top-ranked clusters from MD simulations of alternately-cyclised versions of peptide M7. Backbone is shown as cartoon representation, and non-canonical residues as stick. Atoms are coloured by type (grey, carbon; blue, nitrogen; red, oxygen; yellow, sulfur). (A) Linearised version, with the N-terminus acetylated and the pyridine nitrile replaced with phenylalanine. (B) *Meta*-substituted cross-link, as used in experimental work here. (C) *Para*-substituted cross-link, as previously reported by Iskandar *et al.*^40^ Peptide sequences are as indicated semi-structurally, with ‘B’ as one-letter code for *m*CNP and ‘J’ for *p*CNP.

Peptides with *m*CNP macrocyclisation that were enriched for binding to H1 clearly differed from our previous hits against this same target. This difference presumably arises from the unique conformational constraints imposed by the macrocyclisation, and so we sought to visualise what influence this cross-link has on peptides of this size. We carried out molecular dynamics (MD) simulation of peptide M7, as well as an analogue with a crosslink derived from *para*-substituted pyridine nitrile (*p*CNP) and a linear version (with acetylated N-terminus and *m*CNP substituted to phenylalanine). Input structures for the linear version derived from the iTasser server^37^ showed a strong propensity for beta sheet across the entire length of the peptide, with a turn at Pro9 (Fig. S4†). MD simulations showed this to be stable but with the two termini more dynamic, as would be expected for a short linear peptide (Fig. 5A). Introduction of a crosslink from *m*CNP decreased this terminal flexibility, reinforcing the beta sheet across residues Phe4 through Leu15 (Fig. 5B). The remaining amino acids form an unusual bulged turn structure that appears to be stabilised by hydrophobic interactions of the thiazoline ring with the Phe4 and Leu15 sidechains (Fig. S5†). Changing the crosslinker to *para* rather than *meta* substituted pyridine nitrile had a dramatic effect, disrupting the beta sheet and driving the peptide to a more flexible loop conformation (Fig. 5C), with several hydrophobic sidechains forming a small core. These models indicate that the rigidity of the pyridine–thiazoline crosslink has a strong influence on the conformation of the peptide backbone, it introduces a unique functional group that can engage in stabilising sidechain interactions, and differences in the regiochemistry of the crosslink can lead to large scale differences in peptide conformation. We thus conclude that *m*CNP as a cyclisation moiety can give unique conformational influences on a macrocyclic peptide library, even in a relatively large ring.

### Comparison to alternate approaches

The most common cyclisation approach currently in use for reprogrammed mRNA display is by thioether formation from an N-terminal chloroacetamide to a C-terminal cysteine.^15^ That method suffers from limited regioselectivity if more than one cysteine is present (typically the first in the sequence will react, although that balance can be influenced by choice of initiating amino acid^38^), and also gives a flexible linkage. It is hence incompatible with additional cysteine residues within the macrocycle, which are valuable handles for further bioconjugation or bicyclisation. The more rigid thiazoline that results from the method we describe here, which also has less hydrogen bond donors from the loss of the N-terminal amide NH, has been shown to give improved permeability in a parallel artificial membrane permeability assay (PAMPA) as compared to analogous thioether or amide cyclised peptides.^39^ We consider these two cyclisation approaches to be complementary, giving access to pools of peptides with different conformational influences and so potentially different hits, as demonstrated by our selections against influenza HA. The ability to produce *m*CNP-cyclised peptides by bacterial culture^30^ is a further advantage of our current method here, taking advantage of an aminoacyl tRNA synthetase that can efficiently charge both *p*- and *m*CNP and has been shown to give in-cell peptide macrocyclisation.

Recently published work from the Bowers group showed a related cyclisation using a *para*-substituted pyridine nitrile amino acid.^40^ This was incorporated by the promiscuous aminoacyl-tRNA synthetase *p*-CNF-RS (*p*-cyanophenylalanine specific aminoacyl-tRNA synthetase), which relies on stop codon suppression and is thus less flexible in reprogramming. This nonsense suppression approach risks peptide truncations, and so necessitates an additional purification step using a C-terminal affinity tag that increases the complexity of the workflow. That approach also relied on the enzymes peptide deformylase and methionine aminopeptidase to expose the N-terminal cysteine. These enzymes have their own substrate biases^41,42^ and so are less general than the initiation codon bypass approach we use here. Importantly, the enzyme used in that work, *p*-CNF-RS, was unable to acylate the *meta*-substituted isomer, *m*CNP, described in this work. We again consider both approaches complementary as the *meta*- and *para*-substitution patterns give access to useful differences in peptide conformation, as illustrated by our MD simulations here.

## Conclusions

In this work we have adapted the ‘thiazoline click’ reaction of N-terminal cysteine with *meta*-cyanopyridylalanine for application to *in vitro* translation and subsequently in genetically recoded mRNA-display based peptide selection. We show that this new approach is overall complementary to several existing macrocyclisation options and thereby increases the size of the macrocyclisation toolbox, potentially offering advantages in increased rigidity of the macrocyclisation cross-link, with positive influence on cell permeability; free choice in the genetic recoding for more flexible adjustment of the peptide chemical space; and compatibility with additional cysteine residues, including in spontaneous bicycle formation.

## Data availability

Raw sequencing data have been deposited in the DataverseNL database with https://doi.org/10.34894/GAZQX6.

## Author contributions

ML, DP, CN and SJ designed the research. DP, CH, CN, and SJ supervised the research. ML, RM, RY, MP, AH and ET carried out experimental work. ML, RM, RY, DP, CH, CN and SJ wrote the manuscript.

## Conflicts of interest

There are no conflicts to declare.

## Supplementary Material

SC-014-D3SC03117A-s001

## References

[cit1] Morrison C. (2018). Constrained peptides' time to shine?. Nat. Rev. Drug Discovery.

[cit2] Cary D. R., Ohuchi M., Reid P. C., Masuya K. (2017). Constrained peptides in drug discovery and development. J. Synth. Org. Chem., Jpn..

[cit3] Wang L., Wang N., Zhang W., Cheng X., Yan Z., Shao G., Wang X., Wang R., Fu C. (2022). Therapeutic peptides: current applications and future directions. Signal Transduction Targeted Ther..

[cit4] Zorzi A., Deyle K., Heinis C. (2017). Cyclic peptide therapeutics: past, present and future. Curr. Opin. Chem. Biol..

[cit5] Vinogradov A. A., Yin Y., Suga H. (2019). Macrocyclic Peptides as Drug Candidates: Recent Progress and Remaining Challenges. J. Am. Chem. Soc..

[cit6] Chen S., Morales-Sanfrutos J., Angelini A., Cutting B., Heinis C. (2012). Structurally diverse cyclisation linkers impose different backbone conformations in bicyclic peptides. ChemBioChem.

[cit7] Kale S. S., Villequey C., Kong X.-D. D., Zorzi A., Deyle K., Heinis C. (2018). Cyclization of peptides with two chemical bridges affords large scaffold diversities. Nat. Chem..

[cit8] Li X., Craven T. W., Levine P. M. (2022). Cyclic Peptide Screening Methods for Preclinical Drug Discovery. J. Med. Chem..

[cit9] Alleyne C., Amin R. P., Bhatt B., Bianchi E., Blain J. C., Boyer N., Branca D., Embrey M. W., Ha S. N., Jette K., Johns D. G., Kerekes A. D., Koeplinger K. A., Laplaca D., Li N., Murphy B., Orth P., Ricardo A., Salowe S., Seyb K., Shahripour A., Stringer J. R., Sun Y., Tracy R., Wu C., Xiong Y., Youm H., Zokian H. J., Tucker T. J. (2020). Series of Novel and Highly Potent Cyclic Peptide PCSK9 Inhibitors Derived from an mRNA Display Screen and Optimized *via* Structure-Based Design. J. Med. Chem..

[cit10] Tucker T. J., Embrey M. W., Alleyne C., Amin R. P., Bass A., Bhatt B., Bianchi E., Branca D., Bueters T., Buist N., Ha S. N., Hafey M., He H., Higgins J., Johns D. G., Kerekes A. D., Koeplinger K. A., Kuethe J. T., Li N., Murphy B., Orth P., Salowe S., Shahripour A., Tracy R., Wang W., Wu C., Xiong Y., Zokian H. J., Wood H. B., Walji A. (2021). A Series of Novel, Highly Potent, and Orally Bioavailable Next-Generation Tricyclic Peptide PCSK9 Inhibitors. J. Med. Chem..

[cit11] Huang Y., Wiedmann M. M., Suga H. (2018). RNA Display Methods for the Discovery of Bioactive Macrocycles. Chem. Rev..

[cit12] Goto Y., Katoh T., Suga H. (2011). Flexizymes for genetic code reprogramming. Nat. Protoc..

[cit13] Goto Y., Suga H. (2021). The RaPID Platform for the Discovery of Pseudo-Natural Macrocyclic Peptides. Acc. Chem. Res..

[cit14] Richardson S. L., Dods K. K., Abrigo N. A., Iqbal E. S., Hartman M. C. (2018). In vitro genetic code reprogramming and expansion to study protein function and discover macrocyclic peptide ligands. Curr. Opin. Chem. Biol..

[cit15] Iwasaki K., Goto Y., Katoh T., Suga H. (2012). Selective thioether macrocyclization of peptides having the N-terminal 2-chloroacetyl group and competing two or three cysteine residues in translation. Org. Biomol. Chem..

[cit16] Jongkees S. A. K., Umemoto S., Suga H. (2017). Linker-free incorporation of carbohydrates into *in vitro* displayed macrocyclic peptides. Chem. Sci..

[cit17] Goto Y., Ito Y., Kato Y., Tsunoda S., Suga H. (2014). One-Pot Synthesis of Azoline-Containing Peptides in a Cell-free Translation System Integrated with a Posttranslational Cyclodehydratase. Chem. Biol..

[cit18] Rosen C. B., Francis M. B. (2017). Targeting the N terminus for site-selective protein modification. Nat. Chem. Biol..

[cit19] Asiimwe N., Al Mazid M. F., Murale D. P., Kim Y. K., Lee J.-S. (2021). Recent advances in protein modifications techniques for the targeting N-terminal cysteine. Pept. Sci..

[cit20] Liu M., Thijssen V., Jongkees S. A. K. (2020). Suppression of formylation provides an alternative approach to vacant codon creation in bacterial *in vitro* translation. Angew Chem. Int. Ed. Engl..

[cit21] Nitsche C., Onagi H., Quek J.-P., Otting G., Luo D., Huber T. (2019). Biocompatible Macrocyclization between Cysteine and 2-Cyanopyridine Generates Stable Peptide Inhibitors. Org. Lett..

[cit22] Liu M., Yoshisada R., Amedi A., Hopstaken A. J. P. P., Pascha M. N., de Haan C. A. M., Geerke D. P., Poole D. A., Jongkees S. A. K. (2023). An Efficient, Site-Selective and Spontaneous Peptide Macrocyclisation During *in vitro* Translation. Chem.–Eur. J..

[cit23] Hampton J. T., Lalonde T. J., Tharp J. M., Kurra Y., Alugubelli Y. R., Roundy C. M., Hamer G. L., Xu S., Liu W. R. (2022). Novel Regioselective Approach to Cyclize Phage-Displayed Peptides in Combination with Epitope-Directed Selection to Identify a Potent Neutralizing Macrocyclic Peptide for SARS-CoV-2. ACS Chem. Biol..

[cit24] Zheng M., Haeffner F., Gao J. (2022). N-Terminal cysteine mediated backbone-side chain cyclization for chemically enhanced phage display. Chem. Sci..

[cit25] Fujino T., Kondo T., Suga H., Murakami H. (2019). Exploring of minimal RNA substrate of flexizymes. ChemBioChem.

[cit26] Katoh T., Iwane Y., Suga H. (2018). tRNA engineering for manipulating genetic code. RNA Biol..

[cit27] Cui Z., Wu Y., Mureev S., Alexandrov K. (2018). Oligonucleotide-mediated tRNA sequestration enables one-pot sense codon reassignment *in vitro*. Nucleic Acids Res..

[cit28] Sako Y., Goto Y., Murakami H., Suga H. (2008). Ribosomal synthesis of peptidase-resistant peptides closed by a nonreducible inter-side-chain bond. ACS Chem. Biol..

[cit29] Wendt M., Bellavita R., Gerber A., Efrém N. L., van Ramshorst T., Pearce N. M., Davey P. R. J., Everard I., Vazquez-Chantada M., Chiarparin E., Grieco P., Hennig S., Grossmann T. N. (2021). Bicyclic β-Sheet Mimetics that Target the Transcriptional Coactivator β-Catenin and Inhibit Wnt Signaling. Angew. Chem., Int. Ed..

[cit30] Abdelkader E. H., Qianzhu H., George J., Frkic R. L., Jackson C. J., Nitsche C., Otting G., Huber T. (2022). Genetic Encoding of Cyanopyridylalanine for In-Cell Protein Macrocyclization by the Nitrile–Aminothiol Click Reaction. Angew. Chem., Int. Ed..

[cit31] Pascha M. N., Thijssen V., Egido J. E., Linthorst M. W., Van Lanen J. H., Van Dongen D. A. A., Hopstaken A. J. P. P., Van Kuppeveld F. J. M., Snijder J., De Haan C. A. M., Jongkees S. A. K. (2022). Inhibition of H1 and H5 Influenza A Virus Entry by Diverse Macrocyclic Peptides Targeting the Hemagglutinin Stem Region. ACS Chem. Biol..

[cit32] Ishizawa T., Kawakami T., Reid P. C., Murakami H. (2013). TRAP display: A high-speed selection method for the generation of functional polypeptides. J. Am. Chem. Soc..

[cit33] Evenson W. E., Lin W. Z. S., Pang K., Czaja A. T., Jalali-Yazdi F., Takahashi T. T., Malmstadt N., Roberts R. W. (2020). Enabling Flow-Based Kinetic Off-Rate Selections Using a Microfluidic Enrichment Device. Anal. Chem..

[cit34] Thijssen V., Hurdiss D. L., Debski-Antoniak O. J., Spence M. A., Franck C., Norman A., Aggarwal A., Mokiem N. J., van Dongen D. A. A., Vermeir S. W., Liu M., Li W., Chatziandreou M., Donselaar T., Du W., Drulyte I., Bosch B. J., Snijder J., Turville S. G., Payne R. J., Jackson C. J., van Kuppeveld F. J. M., Jongkees S. A. K. (2023). A broad-spectrum macrocyclic peptide inhibitor of the SARS-CoV-2 spike protein. Proc. Natl. Acad. Sci. U. S. A..

[cit35] Angeletti D., Yewdell J. W. (2018). Is it possible to develop a “universal” influenza virus vaccine?: Outflanking antibody immunodominance on the road to universal influenza vaccination. Cold Spring Harbor Perspect. Biol..

[cit36] Kadam R. U., Juraszek J., Brandenburg B., Buyck C., Schepens W. B. G., Kesteleyn B., Stoops B., Vreeken R. J., Vermond J., Goutier W., Tang C., Vogels R., Friesen R. H. E., Goudsmit J., Van Dongen M. J. P., Wilson I. A. (2017). Potent peptidic fusion inhibitors of influenza virus. Science.

[cit37] Yang J., Zhang Y. (2015). I-TASSER server: New development for protein structure and function predictions. Nucleic Acids Res..

[cit38] Okuma R., Kuwahara T., Yoshikane T., Watanabe M., Dranchak P., Inglese J., Shuto S., Goto Y., Suga H. (2020). A Macrocyclic Peptide Library with a Structurally Constrained Cyclopropane-containing Building Block Leads to Thiol-independent Inhibitors of Phosphoglycerate Mutase. Chem.–Asian J..

[cit39] Tamura T., Inoue M., Yoshimitsu Y., Hashimoto I., Ohashi N., Tsumura K., Suzuki K., Watanabe T., Hohsaka T. (2022). Chemical Synthesis and Cell-Free Expression of Thiazoline Ring-Bridged Cyclic Peptides and Their Properties on Biomembrane Permeability. Bull. Chem. Soc. Jpn..

[cit40] Iskandar S. E., Pelton J. M., Wick E. T., Bolhuis D. L., Baldwin A. S., Emanuele M. J., Brown N. G., Bowers A. A. (2022). Enabling Genetic Code Expansion and Peptide Macrocyclization in mRNA Display *via* a Promiscuous Orthogonal Aminoacyl-tRNA Synthetase. J. Am. Chem. Soc..

[cit41] Xiao Q., Zhang F., Nacev B. A., Liu J. O., Pei D. (2010). Protein N-terminal processing: Substrate specificity of *Escherichia
coli* and human methionine aminopeptidases. Biochemistry.

[cit42] Hu Y. J., Wei Y., Zhou Y., Rajagopalan P. T. R., Pei D. (1999). Determination of substrate specificity for peptide deformylase through the screening of a combinatorial peptide library. Biochemistry.

