# Supplementary material for: Selective thiazoline peptide cyclisation compatible with mRNA display and efficient synthesis
Source: Chem Sci. 2023 Sep 8;14(38):10561–9. doi: 10.1039/d3sc03117a (PMC10548512; doi:10.1039/d3sc03117a)
Supplement: SC-014-D3SC03117A-s001 [file SC-014-D3SC03117A-s001.pdf]

Electronic Supplementary Material (ESI) for Chemical Science.
This journal is © The Royal Society of Chemistry 2023

Supporting information for:

Selective thiazoline peptide cyclisation compatible with mRNA display and efficient synthesis

Minglong Liu,[a,b] Richard Morewood,[c] Ryoji Yoshisada,[a,b] Mirte N. Pascha,[d] Antonius J. P. Hopstaken,[a,b] Eliza Tarcoveanu,[c] David A. Poole III,[a,b] Cornelis A. M. de Haan,[d] Christoph Nitsche,[c*] Seino A. K. Jongkees[a,b*]

[a.] *Department of Chemistry and Pharmaceutical Sciences, Vrije Universiteit Amsterdam, Amsterdam, The Netherlands.*
[b.] *Amsterdam Institute of Molecular and Life Sciences (AIMMS), Vrije Universiteit Amsterdam, Amsterdam, The Netherlands.*
[c.] *Research School of Chemistry, Australian National University, Canberra, ACT 2601, Australia.*
[d.] *Section Virology, Division Infectious Diseases and Immunology, Department of Biomolecular Health Sciences, Faculty of Veterinary Medicine, Utrecht University, Yalelaan 1, 3584 CL Utrecht, The Netherlands.*

General Information

Chemicals were purchased from Sigma Aldrich, Alfa Aesar or GLbiochem and used without further purification. HPLC-grade solvents were purchased from Biosolve. Unless otherwise stated, analytic LC-MS was performed on an Agilent 1260 II Infinity LC system using an Agilent poroshell-120 EC-C18 column (particle size: 2.7 µm, 100 x 4.6 mm) at a flow rate of 0.6 mL.min^{-1} using a linear gradient of buffer A (0.1% Formic acid in H_2O) and buffer B (0.1% Formic acid in CH_3CN) from 5-95% B over 10 min, with detection by UV at 215 and 280 nm and by Agilent InfinityLab LC/MSD XT. Preparative RP-HPLC was performed on an automated preparative HPLC system equipped with a UV/VIS detector at 215/280nm using a C18 column (particle size: 10 µm, 250 x 22 mm) at a flow rate of 12.5 mL.min^{-1} using a linear gradient of buffer A (0.1% TFA in H2O) and buffer B (0.1% TFA in CH_3CN) from 10-70% B over 60 min. Peptide concentration was calculated through the UV absorbance at 280 nm on Nanodrop (Thermo scientific) using extinction coefficients calculated by the Swiss Bioinformatics Institute ProtParam tool and adjusting for pyridylthiazoline (see below). MALDI-TOF mass spectra for translated peptides were measured on Bruker ultrafleXtreme Maldi-TOF-MS and analyzed with flexAnalysis software. U-C18 ZipTip pipette tips (ZTC18M096) were purchased from Merck. Primers were ordered from Integrated DNA Technologies. PURexpress (E6840S and E6850S) was purchased from New England Biolabs. ^1H and ^{13}C NMR spectra were recorded on Bruker 300 MHz, 400 MHz or Bruker 500 MHz systems. All measurements were performed at 25 °C. Spectra were processed using MestReNova. Chemical shifts for all NMR spectra are reported in parts per million (ppm) and were referenced by their residual solvent peaks. Coupling constants (J) are recorded in Hz. Deuterated solvents were purchased from Cambridge Isotope Laboratories (USA). Low-resolution electrospray ionisation mass spectrometry analysis was performed on a Waters LCT Premier orthogonal acceleration time-of-flight mass spectrometer. High-resolution electrospray ionisation mass analysis was performed on a Thermo Scientific Orbitrap Elite mass spectrometer. Analytical thin-layer chromatography analysis was performed on pre-coated silica gel aluminium-backed plates (Merck silica gel 60 F_{254}), using visualisation under UV light (254 nm). Synthesised compounds were purified using a Biotage Isolera One automated flash chromatography system equipped with Biotage SNAP Ultra silica gel cartridges. Elution was monitored by UV absorbance (254 and 280 nm).

Preparation of flexizyme, tRNA, DNA, mRNA

DNA templates were assembled by primer extension followed by PCR. Flexizymes (eFx and dFx), tRNAs ($tRNA^{fMet}_{CAU}$, $tRNA^{EnAsnE2}_{GCU}$, $tRNA^{EnAsnE2}_{CCA}$ and $tRNA^{EnAsnE2}_{CAU}$) and mRNAs were prepared by *in vitro* transcription with T7 RNA polymerase as previously described.[1] A list of primers used for each template is provided in Table S1, how these were assembled is indicated in Table S2, and resulting sequences in Table S3.

Aminoacylation testing

Aminoacylation reactions (5 µL) were carried out with slight modifications from previously reported conditions.[2] A mixture of 3 µL containing 125 pmol of 5b-FAM (5/6-FAM-CGCCA RNA) and 125 pmol dFx or eFx in 0.25 M HEPES-KOH buffer (pH 7.5) was heated at 95 °C for 2 minutes and subsequently allowed to cool to room temperature for 5 minutes. Then, 1 µL 3 M $MgCl_2$ solution was added, followed by an incubation at room temperature for another 5 minutes. The mixture was then cooled on ice, followed by the addition of 1 µL 25 mM amino acid solution in DMSO. The reaction was left to incubate on ice for a varied amount of time (as detailed in the figures). The reaction was quenched with 2X Loading buffer (pH 5.2) and loaded onto 20% acid PAGE gel and run for 150 minutes at 120 V in sodium acetate (pH 5.2) buffer. Gels were subsequently imaged on Azure biosystems C400 and yields were determined by densitometry in ImageJ.

Procedure for aminoacylation of tRNA

Aminoacylation reactions (10 µL) were carried out with the following condition: A mixture of 6 µL of 250 pmol of tRNA and 250 pmol dFx in 0.25 M HEPES-KOH buffer (pH 7.5) was heated at 95 °C for 2 min followed by an incubation at room temperature for 5 minutes. Then, 2 µL $MgCl_2$ 3 M solution was added and the mixture was left to incubate another 5 min at room temperature. The mixture was then cooled on ice, followed by the addition of 2 µL amino acid 25 mM solution in DMSO. The reaction was left to incubate on ice for 2 hours then quenched with 40 µL NaOAc buffer (0.3 M, pH 5.2). This was followed by the addition of 100 µL ethanol to precipitate the product, and the mixture was then centrifuged at 4 °C 15000 rcf for 15 min to form a pellet of the precipitated product. The supernatant was removed and the pellet was vortexed with 40 µL NaOAc buffer (0.1 M, pH 5.2) in 70% EtOH then centrifuged for 10 minutes at 15000 rcf, and this process was repeated once more for a total of two washes. A final wash was then performed (without vortexing) using 30 µL 70% ethanol in water and followed by a 3 min centrifugation at 15000 rcf at 4 °C. Following removal of the supernatant, the pellet was left to air dry for 5 minutes and stored at -20 °C.

Procedure for translation and MALDI-TOF mass test

Translation of **P1**, **P2**, **P3** and **P4** was carried out using PURExpress *in vitro* translation system (New England Biolabs, USA) based on an adjusted version of the manufacturer's condition at 37 °C for 30 min. Solution A of the kit was replaced with a home-made version containing 50 mM HEPES-KOH (pH 7.6), 100 mM potassium acetate, 12 mM magnesium acetate, 2 mM ATP, 2 mM GTP, 1 mM CTP, 1 mM UTP, 20 mM creatine phosphate, 2 mM spermidine, 1 mM DTT, 1.5 mg/mL E. coli total tRNA, 50 µM $tRNA^{fMet}$ (uncharged), 50 µM mCNP-$tRNA^{EnAsnE2}_{GCU}$ (**P1**, **P2)** or 50 µM mCNP-$tRNA^{EnAsnE2}_{CAU}$ (**P3**, **P4**), 0.5 mM amino acids mix (omitting methionine), 0.1 µM DNA, 10 µM M5 suppressor (**P1**, **P2**).[3] After translation, EDTA was added to 10 mM and the solution further incubated for 1.5 hrs at the same temperature. Samples were analysed by pre-purification through C18

extraction in microtip format, washing with 4% acetonitrile in water and eluting in 80% (both with 0.1% formic acid) before spotting on MALDI plates with 70% saturated α-CHCA in the elution solvent.

The translation of **P5** was carried as the described above except that 50 μM of ClAcDap-tRNA$^{EnAsnE2}_{CCA}$ was added and tryptophan was also omitted from the amino acid mix. After 30 min translation and addition of EDTA, 2X cyclization buffer (0.1 M Tris, pH 8.3, 1.4 M NaCl) was added to the sample and the mixture was allowed to incubate at 37 °C for 30 min before analysis by MALDI-TOF-MS as described above. Peptide **P6** was translated as for **P5**, but with aminoacylation of ClAcDap and mCNP on GCU and CCA codons (respectively).

In vitro selection of macrocyclic peptides by using TRAP display

The selection of peptides followed the protocol described before[4]: the translation mixture (10 μL for 1st round and 5 μL for 2nd and later rounds) was prepared with a homemade solution A described above and solution B from the PURExpress Δ RF123 kit (New England Biolabs, USA), with 11 μM TRAP-Pu linker,[5] 50 μM tRNA^{fMet} (uncharged), and 50 μM mCNP-tRNA$^{EnAsnE2}_{CAU}$. The translation was incubated at 37 °C for 30 min, then 1 μL 100 mM $MgCl_2$ 1 M KCl solution was added and incubated at 37 °C for a further 1 hr, followed by addition of of 2.25 μL 100 mM EDTA. To this translation mixture was added 8.8 μL of 2.5X RT mix (0.625 mM dNTPs, 5 μM G2AGAS.R20, 62.5 mM Tris.HCl pH 8.3, 37.5 mM $MgCl_2$, 25 mM KOH, 2.5X MMLV reverse transcriptase) and the solution was incubated at 42 °C for 1.5 hr. Acetylated bovine serum albumin was added (to a final concentration of 1 mg/mL) and the solution was incubated in the presence of MagStrep "Type 3" Strep-Tactin™ (IBA-lifesciences, Germany) beads to remove bead-binding macrocyclic peptides. 1 μg of HA protein (H1 derived from A/California/04/2009, H1N1) was immobilized on Strep-Tactin™ beads by incubation at 4°C for 1 hr, then washed three times with PBS-T (20mM Phosphate pH 7.4, 140 mM NaCl, 0.02% Tween-20). The library solution was incubated with these protein-immoblized beads at 4°C for 30 min to isolate the HA protein binders. The beads were washed 3 times with PBS-T buffer and the peptide-mRNA/cDNA was released by incubating in 50 μL milliq water at 95°C for 5 min. 1 μL of the sample was used for quantification of recovered cDNA by qPCR alongside a standard curve of input library mRNA/cDNA prepared by reverse transcription under the same conditions as above, and the rest of the eluted cDNA was used for PCR (95 °C for 40 s, 61 °C for 40 s, 72 °C for 40 s) after adding of 2X KOD buffer (240 mM Tris.HCl pH 8.0, 20 mM KCl, 12 mM ammonium sulfate, 0.2% Triton X-100 v/v, 0.002% bovine serum albumin w/v) and KOD polymerase enzyme. The resulting solution was used in the next round of selection without further purification. Following selection, all rounds were sequenced by ISeq (Illumina, USA) and data processed as previously described.[6]

Flow test from 4th round selection cDNA

The library solution prepared as above was incubated with HA protein immobilised beads at 4°C for 30 min to isolate the binders. After incubation, the beads were split into 2 portions. One portion was washed 3 times following the standard protocol above, another portion was washed by flow of PBS-T at 200 μl.min^{-1} by syringe pump for 15 min at 4°C with beads retained on a 10 μm frit (Screening Devices B.V., Netherlands, product OC-803) mounted in a custom-milled water-tight holder. All further steps were as above.

UV absorption of pyridyl-thiazoline

A sample of pyridyl-thiazoline was prepared by reaction of 5 mM 3-(2-cyano-4-pyridyl)-alanine with 2 equivalents of free cysteine for 2 hours at room temperature. This gave a molar extinction coefficient of 4950 $M^{-1}.cm^{-1}$ at 280 nm by measurement of absorbance of a sample in 20 mM phosphate buffer with 140 mM NaCl at pH 7.5 on a Nanodrop spectrophotometer (Thermo Scientific, USA). This value was stable after overnight incubation.

Chemical synthesis of peptides

The peptide was synthesized by standard Fmoc solid phase peptide synthesis using a PurePep Chorus automated peptide synthesizer (Gyros protein technologies, Sweden). Fmoc-L-3-(2-cyano-4-pyridyl)-alanine was synthesized as previously described.[7] Each Fmoc-amino acid was coupled for 15 min at 55 °C on TG XV RAM resin (Rapp Polymere, Germany) with 10 equivalents of DIC and 5 equivalents of Oxyma. After coupling, any unreacted peptide was capped with 2 M each acetic anhydride and pyridine in DMF for 5 min, then the *N*-terminus was deprotected with 20% piperidine/0.1 M Oxyma in DMF at room temperature for 10 min. Fmoc-mCNP-OH[7] coupling was carried with 1.5 equivalents of Fmoc building block, 4 equivalents of DIC and 4 equivalents of Oxyma at room temperature overnight. After synthesis, the resin was washed with DMF, DCM and dried under vacuum, then peptide was cleaved off and globally deprotected by swelling dry resin with TFA/H_2O/TIPS (92.5/5/2.5) and incubating for 3 hours. Peptide was precipitated by addition of the TFA solution to 10 volume excess of ice-cold diethyl ether and pelleted by centrifugation at 5k rcf for 10 min, then the crude peptide was redissolved in 1 mL DMSO. The solution was then added into 1 mL 100 mM tris buffer (pH 7.5) and 4 mM TCEP and incubated at room temperature for 1 hr, following the cyclisation by LC-MS, before purification by RP-HPLC. The peptide concentration was quantified by calculated extinction coefficient at A_{280}.

Virus neutralization assays

Virus neutralization by macrocyclic peptides was assessed using a luciferase reporter assay as previously described.[4] Peptides were measured in duplicate using 1 μM peptide for an initial screen, and then for active hits with a dilution series starting from 1 μM with three biological replicates.

In short, this assay measures infection of HeLa R19 cells by virus, with infection triggering luciferase expression. Cells are transfected with a pHH-Gluc luciferase reporter plasmid, which contains the Gaussian luciferase gene flanked by untranslated regions of the IAV NP genome segment and under the control of the RNA polymerase I promoter in the negative sense orientation. Synthesis of mRNA and thus of luciferase protein is triggered by introduction of viral polymerase following infection, which is assayed in the cell supernatant with a Renilla luciferase assay kit following manufacturer's instructions (Promega). Luminescence data were fit using GraphPad Prism 9.1.0 to derive half-maximum inhibitor concentrations (IC_{50}).

Fluorescence polarization

Fluorescence polarization competition assays were performed in black, low-volume, nonbinding 384 well microplates "784900" (Greiner Bio-One, the Netherlands) using a BMG Labtech (Germany) PHERAstar FS microplate reader. The assay buffer consisted of 10 mM Tris.HCl pH 7.5, 150 mM NaCl, 1% DMSO v/v and 0.01% Tween-20 v/v. Peptides were titrated in triplicate to 10 nM H1 ectodomain by performing a 1:1 serial dilution. After 10 min of incubation at room temperature, S5-FAM probe[4] was added 1:1 to each well to reach a final concentration of 5 nM H1 ectodomain and 5 nM S5-FAM. Measurements were performed after another 30 min incubation at room temperature (Excitation: 485 nm, EmissionA: 520 nm, EmissionB: 520 nm). Data were fit using GraphPad Prism 8.4.3 software to the following equation:

$$Y = bottom + \frac{top - bottom}{1 + \left(\frac{IC_{50}}{X}\right)^{hillslope}}$$

Molecular dynamics simulations of peptide M7 and variants

Parametrisation of meta-CNP and para-CNP were performed with DFT at a B3LYP/def2SVP level of theory using Gaussian 16, revision D. The resulting DFT densities were used for RESP fitting to produce models of cyclisation motifs with antechamber following standard procedures (Figure S6 and S7).[8]

Predicted structures of H-CLYFPYFVPNGILQFG-OH were prepared with I-TASSER.[9] Each model was modified with amide on its *C*-terminus, then for linear peptide, the *N*-terminus was capped with acetyl group Ac-CLYFPYFVPNGILQFG-NH_2, while for CNP cyclisation, the peptide was modified to meta-CNP cyclic peptide cyclo[CLYFPYFVPNGILQB]G-NH_2 (where B indicates mCNP) and para-CNP cyclic peptide cyclo[CLYFPYFVPNGILQJ]G-NH_2 (where J indicates pCNP).

Molecular dynamics simulations were carried out using CUDA-enabled Amber22 software (Table S5).[10] Models were firstly minimised in implicit water using the Generalized Born (GB) implicit solvent model with the OBC(II) parameters, then heated up to 300 K over 10 psec. The productive simulation was carried out at 300 K over 50 ns with the timestep of 1 fs. The non-bonded interactions were treated using switching function for van der Waals interactions, and bond constraints involving hydrogen atoms were maintained using the SHAKE algorithm. The Langevin dynamics was used for temperature control with the collision frequency at 10 fs. The trajectories of two models were (k-means) conformationally clustered to produce10 clusters.

Chemical synthesis of activated amino acids

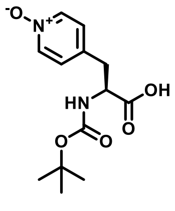

(*S*)-4-(2-((tert-butoxycarbonyl)amino)-2-carboxyethyl)pyridine 1-oxide

A 250 ml round bottom flask was charged with (*S*)-2-((tert-butoxycarbonyl)amino)-3-(pyridin-4-yl)propanoic acid (2.00 g, 7.51 mmol) and 3-chlorobenzoperoxoic acid (1.85 g, 77%, 8.26 mmol) in DCM (100 ml). The mixture was stirred for 18 h at rt. The solvent was removed under reduced pressure and the crude product was purified using a 100 g silica gel cartridge (0 - 10% MeOH:DCM + 0.5% AcOH) to yield the title compound as a white powder (1.86 g, 88%). **^{1}H NMR** (400 MHz, CD_3OD) δ 8.27 (d, J = 6.8 Hz, 2H), 7.46 (d, J = 6.4 Hz, 2H), 4.42 (dd, J = 9.6, 4.9 Hz, 1H), 3.30 – 2.94 (m, 2H), 1.38 (s, 9H) ppm; **^{13}C NMR** (100 MHz, CD_3OD) δ 174.3, 157.7, 143.3, 139.9, 129.0, 80.7, 55.2, 37.4, 28.6 ppm; **LRMS** (ESI^+) *m/z*: 283.1 $[M+H]^+$; **HRMS** (ESI^+) *m/z*: $[M+H]^+$ Calcd for $C_{13}H_{18}N_2O_5$ 283.1294, Found 283.1294; **TLC** R_f: 0.15 (10% MeOH:DCM).

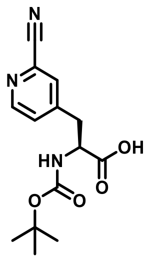

(*S*)-2-((*Tert*-butoxycarbonyl)amino)-3-(2-cyanopyridin-4-yl)propanoic acid

A 500 ml round bottom flask was charged with (*S*)-4-(2-((tert-butoxycarbonyl)amino)-2-carboxyethyl)pyridine 1-oxide (1.86 g, 6.59 mmol), dimethylcarbamic chloride (1.82 ml, 19.8 mmol), and trimethylsilanecarbonitrile (2.47 ml, 19.8 mmol) in DCM (200 ml). The mixture was stirred for 2 days at rt. The solvent was removed under reduced pressure and the crude product was purified using a 100 g silica gel cartridge (0 - 5% MeOH:DCM + 0.5% AcOH) to yield the title compound as a white powder (1.57 g, 82%). ^{1}**H NMR** (400 MHz, acetone-d6) δ 8.65 (d, J = 5.0 Hz, 1H), 7.87 (s, 1H), 7.65 (d, J = 4.3 Hz, 1H), 6.33 (d, J = 8.1 Hz, 1H), 4.56 (td, J = 9.3, 4.8 Hz, 1H), 3.44 – 3.06 (m, 2H), 1.34 (s, 9H) ppm; ^{13}**C NMR** (100 MHz, acetone-d6) δ 172.7, 156.2, 151.8, 150.1, 134.3, 130.6, 129.3, 118.3, 79.6, 54.4, 37.3, 28.4 ppm; **LRMS** (ESI$^+$) *m/z*: 292.1 [M+H]$^+$; **HRMS** (ESI$^+$) *m/z*: [M+H]$^+$ Calcd for $C_{14}H_{17}N_3O_4$ 292.1297, Found 292.1298; **TLC** R_f: 0.28 (20% MeOH:DCM).

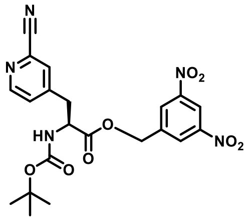

3,5-Dinitrobenzyl (*S*)-2-((*tert*-butoxycarbonyl)amino)-3-(2-cyanopyridin-4-yl)propanoate

A 250 ml round bottom flask was charged with (*S*)-2-((*tert*-butoxycarbonyl)amino)-3-(2-cyanopyridin-4-yl)propanoic acid (300 mg, 1.03 mmol), 1-(chloromethyl)-3,5-dinitrobenzene (446 mg, 2.06 mmol), and DIPEA (538 µl, 3.09 mmol) in DCM (100 ml). The mixture was stirred for 2 days at rt. The mixture was washed with water, brine, dried over Na_2SO_4, and filtered. The solvent was removed under reduced pressure and the crude product was purified using a 10 g silica gel cartridge (0 - 1% MeOH:DCM) to yield the title compound as a white foam (272 mg, 56%). ^{1}**H NMR** (400 MHz, $CDCl_3$) δ 9.02 (t, J = 2.1 Hz, 1H), 8.61 (d, J = 5.1 Hz, 1H), 8.53 (d, J = 2.1 Hz, 2H), 7.51 (s, 1H), 7.37 (dd, J = 5.0, 1.7 Hz, 1H), 5.37 (app dd, J = 29.6, 13.3 Hz, 2H), 5.14 (d, J = 8.2 Hz, 1H), 4.67 (app q, J = 6.8 Hz, 1H), 3.34 – 3.02 (m, 2H), 1.39 (s, 9H) ppm; ^{13}**C NMR** (100 MHz, $CDCl_3$) δ 170.7, 155.0, 151.3, 148.9, 147.2, 139.3, 134.2, 129.4, 128.2, 127.9, 119.1, 117.1, 81.2, 65.2, 53.7, 37.4, 28.3 ppm; **LRMS** (ESI$^+$) *m/z*: 510.0 [M+K]$^+$; **HRMS** (ESI$^+$) *m/z*: [M+H]$^+$ Calcd for $C_{21}H_{21}N_5O_8$ 472.1468, Found 472.1469; **TLC** R_f: 0.32 (2% MeOH:DCM).

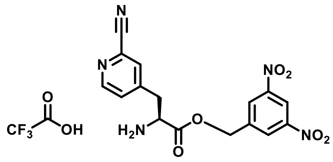

3,5-Dinitrobenzyl (*S*)-2-amino-3-(2-cyanopyridin-4-yl)propanoate trifluoroacetate

A 100 ml round bottom flask was charged with 3,5-dinitrobenzyl (*S*)-2-((*tert*-butoxycarbonyl)amino)-3-(2-cyanopyridin-4-yl)propanoate (200 mg, 424 µmol) in DCM (10 ml). TFA (10 ml) was slowly added, and the mixture was stirred at rt for 1 h. The solvent was removed under reduced pressure and the crude product was purified using a 10 g silica gel cartridge (0 - 5% MeOH:DCM) followed by a second 10 g silica gel cartridge (0 - 7% MeOH:toluene) to yield the title compound as a brown powder (92.8 mg, 45%). ^{1}**H NMR** (400 MHz, CD_3OD) δ 8.98 (t, J = 2.0 Hz, 1H), 8.59 (s, 1H), 8.57 (d, J = 2.1 Hz, 2H), 7.79 (s, 1H), 7.60 (dd, J = 5.1, 1.4 Hz, 1H), 5.45 (app dd, J = 18.3, 13.0 Hz, 2H), 4.55 (t, J = 7.3 Hz, 1H), 3.41 – 3.25 (m, 2H) ppm; ^{13}**C NMR** (100 MHz, CD_3OD) δ 170.0, 152.4, 150.0, 148.0, 140.5, 134.9, 130.8, 129.6, 129.5, 119.7, 117.9, 66.9, 54.0, 36.7 ppm; **LRMS** (ESI$^+$) *m/z*: 372.1 [M+H]$^+$; **HRMS** (ESI$^+$) *m/z*: [M+H]$^+$ Calcd for $C_{16}H_{13}N_5O_6$ 372.0944, Found 372.0946; **TLC** R_f: 0.10 (10% MeOH:toluene).

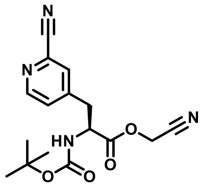

Cyanomethyl (*S*)-2-((*tert*-butoxycarbonyl)amino)-3-(2-cyanopyridin-4-yl)propanoate

A 50 ml round bottom flask was charged with (*S*)-2-((*tert*-butoxycarbonyl)amino)-3-(2-cyanopyridin-4-yl)propanoic acid (300 mg, 1.03 mmol) and DIPEA (538 µl, 3.09 mmol) in chloroacetonitrile (5 ml). The mixture was stirred for 2 days at rt. The mixture was washed with water, brine, dried over Na_2SO_4, and filtered. The solvent was removed under reduced pressure and the crude product was purified using a 10 g silica gel cartridge (0 - 2% MeOH:DCM) to yield the title compound as a yellow solid (259 mg, 76%). **^{1}H NMR** (400 MHz, $CDCl_3$) *δ* 8.66 (d, *J* = 4.9 Hz, 1H), 7.55 (s, 1H), 7.38 (dd, *J* = 5.0, 1.7 Hz, 1H), 5.11 (d, *J* = 7.4 Hz, 1H), 4.80 (app dd, *J* = 41.8, 15.6 Hz, 2H), 4.68 (td, *J* = 14.4, 6.8 Hz, 1H), 3.34 – 3.03 (m, 2H), 1.41 (s, 9H) ppm; **^{13}C NMR** (100 MHz, $CDCl_3$) *δ* 169.8, 155.0, 151.4, 146.8, 134.3, 129.4, 127.9, 117.1, 113.7, 81.4, 53.4, 49.4, 37.4, 28.3 ppm; **LRMS** (ESI$^+$) *m/z*: 353.1 $[M+Na]^+$; **HRMS** (ESI$^+$) *m/z*: $[M+H]^+$ Calcd for $C_{16}H_{18}N_4O_4$ 331.1406, Found 331.1406; **TLC** R_f: 0.21 (2% MeOH:DCM).

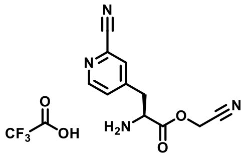

Cyanomethyl (*S*)-2-amino-3-(2-cyanopyridin-4-yl)propanoate trifluoroacetate

A 100 ml round bottom flask was charged with cyanomethyl (*S*)-2-((*tert*-butoxycarbonyl)amino)-3-(2-cyanopyridin-4-yl)propanoate (200 mg, 605 µmol) in DCM (10 ml). TFA (10 ml) was slowly added, and the mixture was stirred at rt for 1 h. The solvent was removed under reduced pressure and the residue was purified by RP-HPLC (C18Aq, 0 - 10% MeOH:water + 0.1% TFA) to yield the title compound as a yellow solid (20.9 mg, 10%). **^{1}H NMR** (400 MHz, CD_3CN) *δ* 8.65 (d, *J* = 5.1 Hz, 1H), 7.82 (s, 1H), 7.59 (d, *J* = 5.0 Hz, 1H), 4.86 (s, 2H), 4.47 (t, *J* = 6.9 Hz, 1H), 3.37 (d, *J* = 7.0 Hz, 2H) ppm; **^{13}C NMR** (100 MHz, CD_3CN) *δ* 168.5, 152.4, 146.6, 134.7, 130.9, 129.6, 118.3, 115.3, 53.9, 51.3, 35.4 ppm; **LRMS** (ESI$^+$) *m/z*: 231.1 $[M+H]^+$; **HRMS** (ESI$^+$) *m/z*: $[M+H]^+$ Calcd for $C_{11}H_{10}N_4O_2$ 231.0882, Found 231.0881; **TLC** R_f: 0.20 (5% MeOH:DCM).

Tables

Table S1. Primers used.

Primer name	Sequence (5'-3')
T7g10M.F48	taatacgactcactatagggttaactttaagaaggagatatacatatg
2CGKR.R50	taccaggtgctcggaaacagaatgcacatatgtatatctccttcttaaag
CGKR.R49	tttccgcccccgtcctagcgtttgcccacataccaggtgctcggaaac
M2_10.R49	gtttgccggtcggaaacagaatgcacatatgtatatctccttcttaaag
M2_10_S.R43	tttccgcccccgtcctagctgcgtttgccggtcggaaacag
M2C_C14_S.F60	ctttaagaaggagatatacatatgtgcattctgtttccgtgcacctggtatgtgggcgcg
C14_S.R47	tttccgcccccgtcctagcctttgctgcccgcgcccacataccagg
M2C_W.F54	ctttaagaaggagatatacatatgtgcatttggtttatgaccggcaaacgcagc
W_S.R34	tttccgcccccgtcctagctgcgtttgccggtc
M2C_M.F55	ctttaagaaggagatatacatatgtgcaacaccccgggccagaaactgtattttc
M2C_M.R52	tttccgcccccgtcctagctttccatcgcatgaaaatacagtttctggccc
T7SD8M2.F44	atactaatacgactcactataggattaaggaggtgatatttatg
MCNNK15MGAS.R90	ctcgctcctgctccacccatmnnmnnmnnmnnmnnmnnmnnmnnmnnmnnmnnmnnmnnmnnmnngcacataaatatcacctccttaatc
G2AGASan.R41	cccgcctcccgcccccgtcctagctcgctcctgctccacc

Table S2. DNA template assembly by PCR.

DNA	Extension		PCR	
Template	Forward	Reverse	Forward	Reverse
M2CGKR	T7g10M.F48	2CGKR.R50	T7g10M.F48	CGKR.R49
M2_10_S	T7g10M.F48	M2_10.R49	T7g10M.F48	M2_10_S.R43
M2_W_S	M2C_W.F54	W_S.R34	T7g10M.F48	W_S.R34
M2C_C14_S	M2C_C14.F60	C14_S.R47	T7g10M.F48	C14_S.R47
M2C_M	M2C_M.F55	M2C_M.R52	T7g10M.F48	M2C_M.R52
mCNP-NNK15	T7SD8M2.F44	MCNNK15MGAS.R90	T7SD8M2.F44	G2AGASan.R41

Table S3. Template sequences.

Peptide name	DNA Template	DNA sequence	Canonical peptide sequence
**P1**	M2_10_S	taatacgactcactatagggttaactttaagaaggagatatacatatgtgcattctgtttccgaccggcaaacgca gctaggacggggggcggaaa	MCILFPTGKRS
**P2**	M2CGKR	taatacgactcactatagggttaactttaagaaggagatatacatatgtgcattctgtttccgagcacctggtatgtg ggcaaacgctaggacggggggcggaaa	MCILFPSTWYVGKR
**P3**	M2_W_S	taatacgactcactatagggttaactttaagaaggagatatacatatgtgcatttggtttatgaccggcaaacgca gctaggacggggggcggaaa	MCIWFMTGKRS
**P4**	M2C_M	taatacgactcactatagggttaactttaagaaggagatatacatatgtgcaacaccccgggccagaaactgtat tttcatgcgatggaaagctaggacggggggcggaaa	MCNTPGQKLYFHAMES
**P5/P6**	M2C_C14_S	taatacgactcactatagggttaactttaagaaggagatatacatatgtgcattctgtttccgtgcacctggtatgtg ggcgcgggcagcaaaggctaggacggggggcggaaa	MCILFPCTWYVGAGSKG
library	mCNP-NNK15	atactaatacgactcactataggattaaggaggtgatatttatgtgcnnknnknnknnknnknnknnknnknn knnknnknnknnknnknnkatgggtggagcaggagcgagctaggacggggggcgggaggcggg	MCXXXXXXXXXXXXXXX MGGAGAS

Table S4. Peptides from SPPS. 'B' indicates mCNP.

name	Linear sequence	Calculated mass $[M+3H]^{3+}$	Observed mass
**M1**	H-CYLIFHQLFSTTLSIRBG-NH_2	718.7	718.8
**M2**	H-CIACWIEGSFKLIFKRBG-NH_2	709.4	709.4
**M3**	H-CLNFQYRRSCILNILNBG-NH_2	728.0	728.1
**M4**	H-CLNFQYRRSCILNILNBG-NH_2	733.4	733.4
**M5**	H-CLFVFNWPDGGSITNKBG-NH_2	670.6	670.7
**M6**	H-CAYTLTRWPYLRLQNLBG-NH_2	741.7	741.8
**M7**	H-CLYFPYFVPNGINILEBG-NH_2	705.3	705.3
**M8**	H-CYYFTHSIFNNLIVFHBG-NH_2	744.0	744.1

Table S5. pmemd.cuda input files used for energy minimisation (left), heating (mid) and productive simulation (right). All calculations were GPU-accelerated.

**Min.in**	**Heat.in**	**Prod.in**
Minimise<br>&cntrl<br>imin=1<br>ntx=1<br>irest=0<br>maxcyc=2000<br>ncyc=1000<br>ntpr=100<br>ntwx=0<br>cut=9999<br>ntb=0<br>igb=5<br>/	Heat<br>&cntrl<br>imin=0,<br>ntx=1,<br>irest=0,<br>nstlim=10000,<br>dt=0.001,<br>ntf=2,<br>ntc=2,<br>tempi=0.0,<br>temp0=300.0,<br>ntpr=100,<br>ntwx=100,<br>cut=9999,<br>ntb=0,<br>igb=5,<br>ntp=0,<br>ntt=3,<br>gamma_ln=0.1,<br>nmropt=1,<br>ig=-1,<br>/<br>&wt type='TEMP0', istep1=0, istep2=9000, value1=0.0, value2=300.0 /<br>&wt type='TEMP0', istep1=9001, istep2=10000, value1=300.0, value2=300.0 /<br>&wt type='END' /	Production<br>&cntrl<br>imin=0,<br>ntx=5,<br>irest=1,<br>nstlim=50000000,<br>dt=0.001,<br>ntf=2,<br>ntc=2,<br>temp0=300.0,<br>ntpr=25000,<br>ntwx=25000,<br>cut=9999,<br>ntb=0,<br>igb=5,<br>ntt=3,<br>gamma_ln=0.01,<br>ig=-1,<br>/

Figures

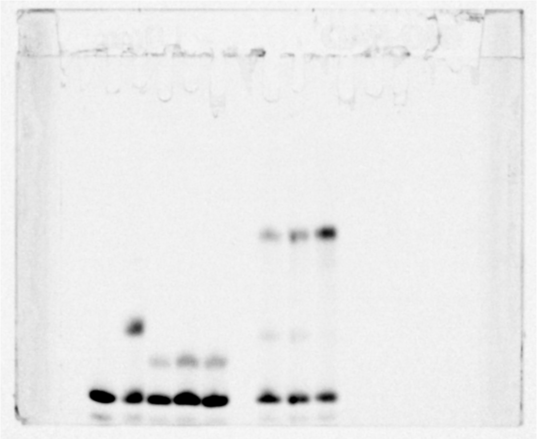

Figure S1. Whole gel image for mCNP-dinitrobenzyl ester (left set of lanes)

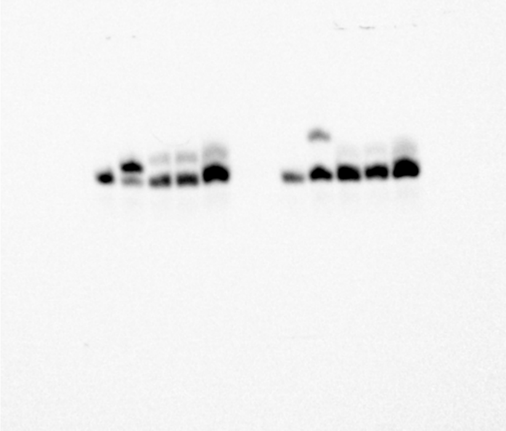

Figure S2. Whole gel image for mCNP-cyanomethyl ester (left set of lanes)

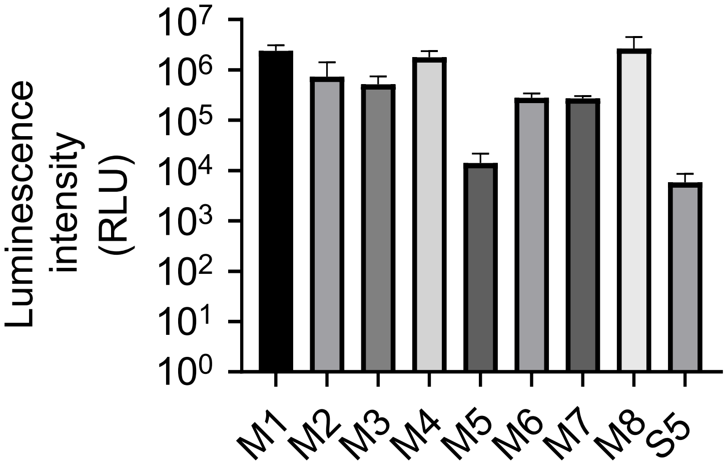

Figure S3. Screen for peptide inhibition activity at 1 µM peptide with H1N1pdm09 virus (A/Netherlands/602/2009) I375F mutant in a luciferase reporter assay. S5 is a positive control thioether-macrocyclised peptide inhibitor from previous work.

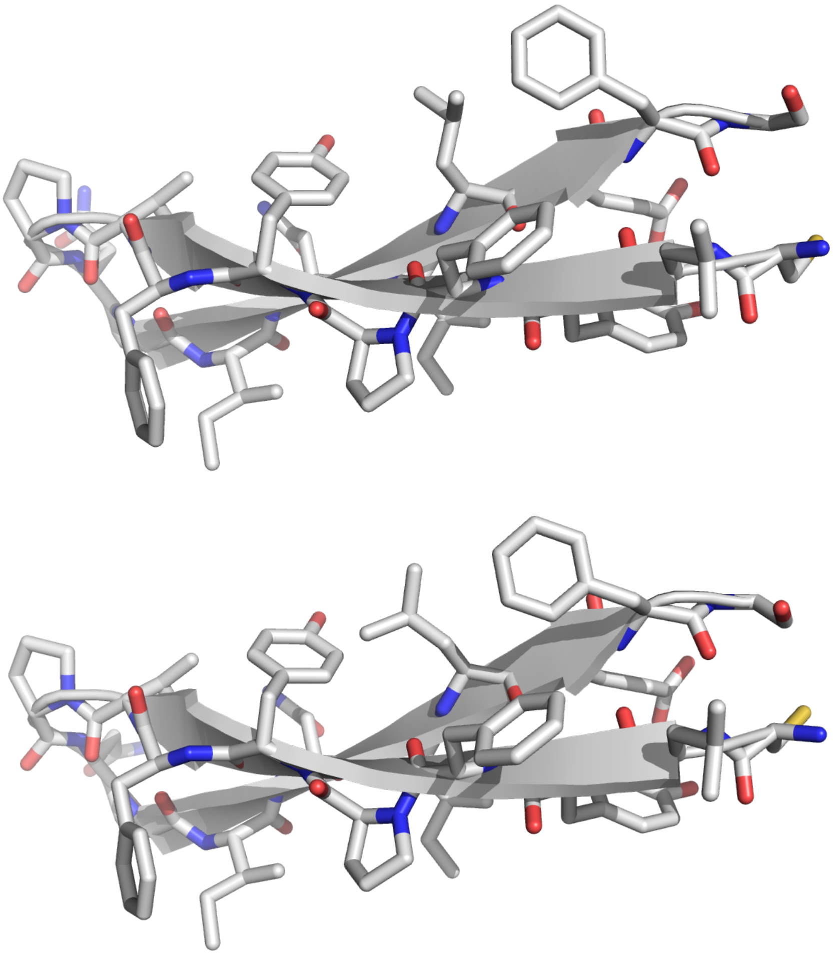

Figure S4. Input structures for MD simulations, derived from the top two output clusters from the iTasser server using linear canonical peptide sequence H-CLYFPYFVPNGINILEFG-OH as input.

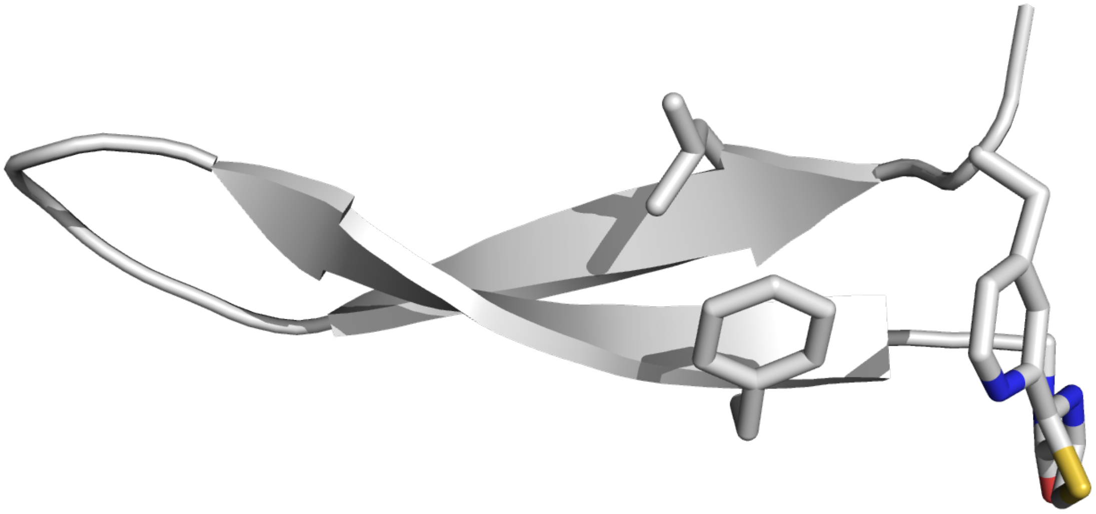

Figure S5. Hydrophobic interactions of mCNP with side chains.

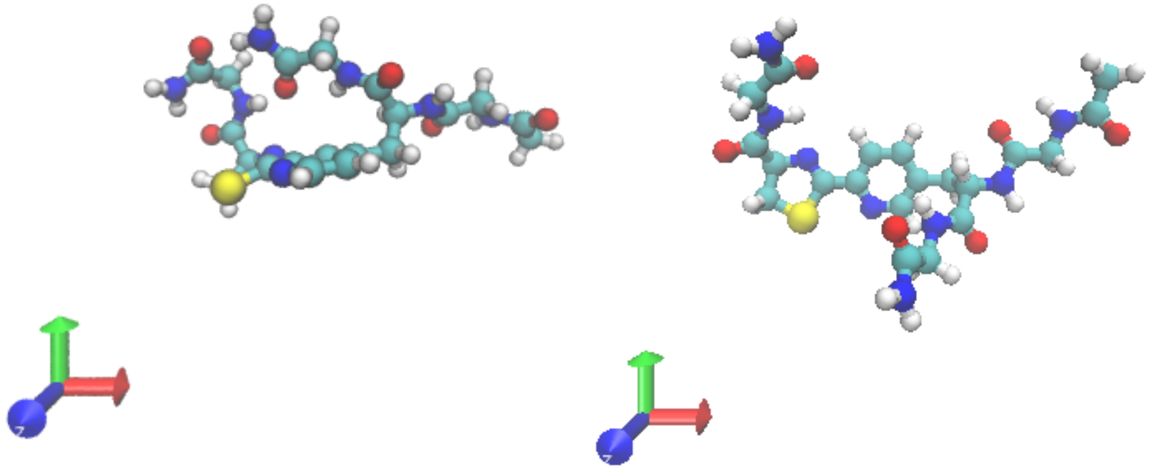

Figure S6. Models used for parameterisation by DFT calculations.

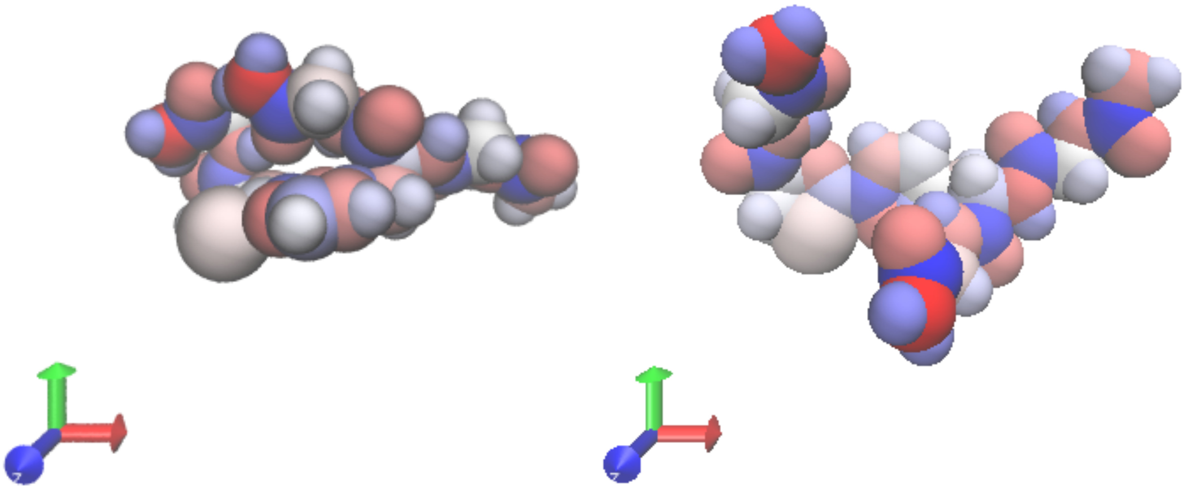

Figure S7. Charge assignments produced by DFT calculations for meta CNP and para CNP cyclisation residues (left and right, respectively). Red represents negative charges and blue represents positive charges.

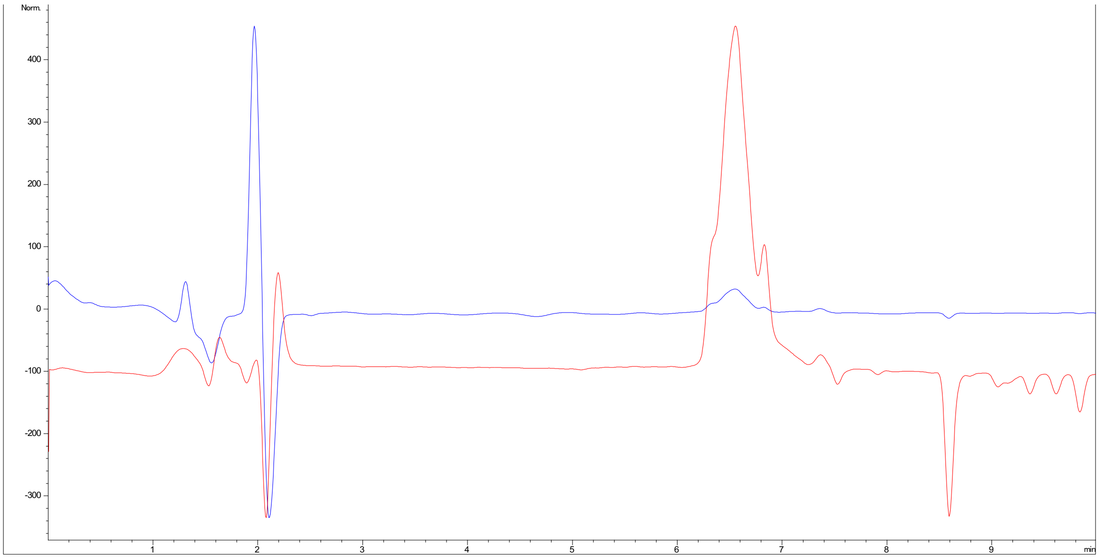

Figure S8. M1 HPLC trace, showing A215 in red and A280 in blue (Y-axis shown for A215, A280 trace autoscaled to fit).

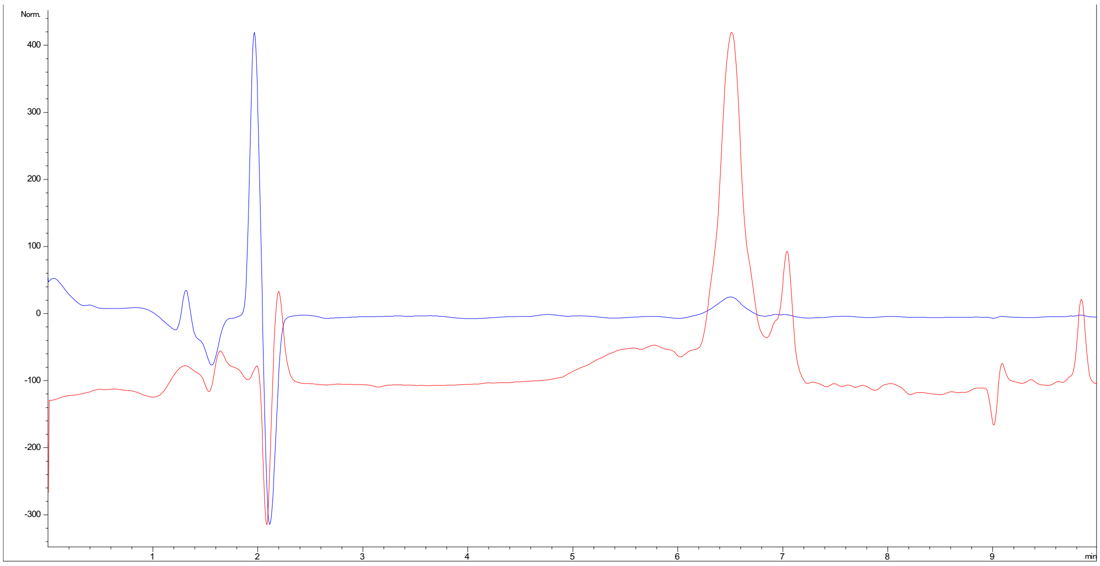

Figure S9. M2 HPLC trace, showing A215 in red and A280 in blue (Y-axis shown for A215, A280 trace autoscaled to fit).

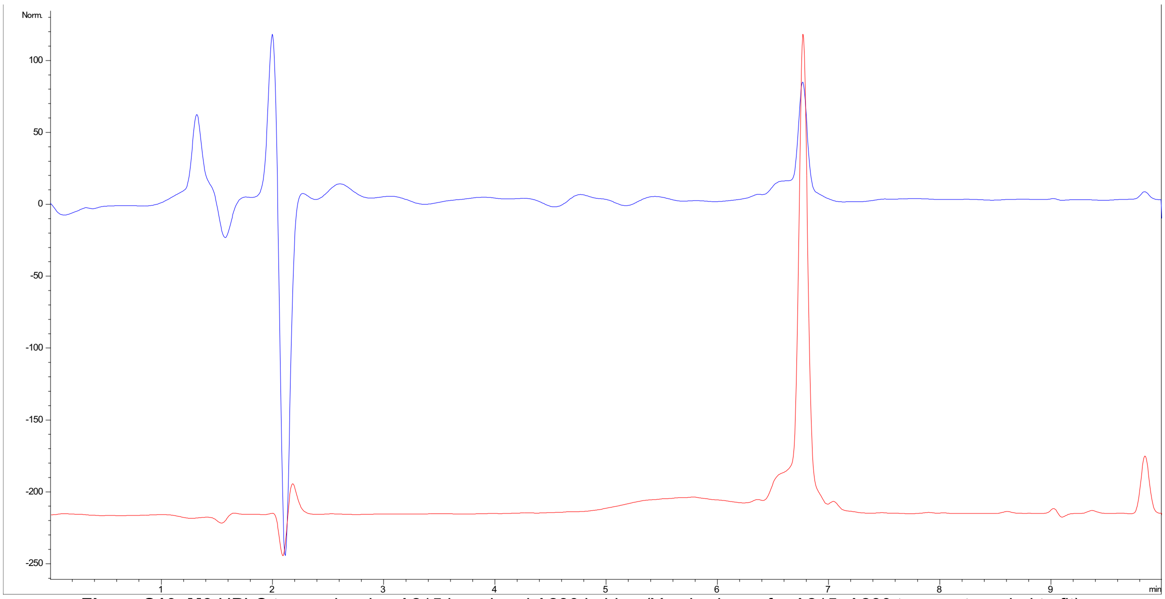

Figure S10. M3 HPLC trace, showing A215 in red and A280 in blue (Y-axis shown for A215, A280 trace autoscaled to fit).

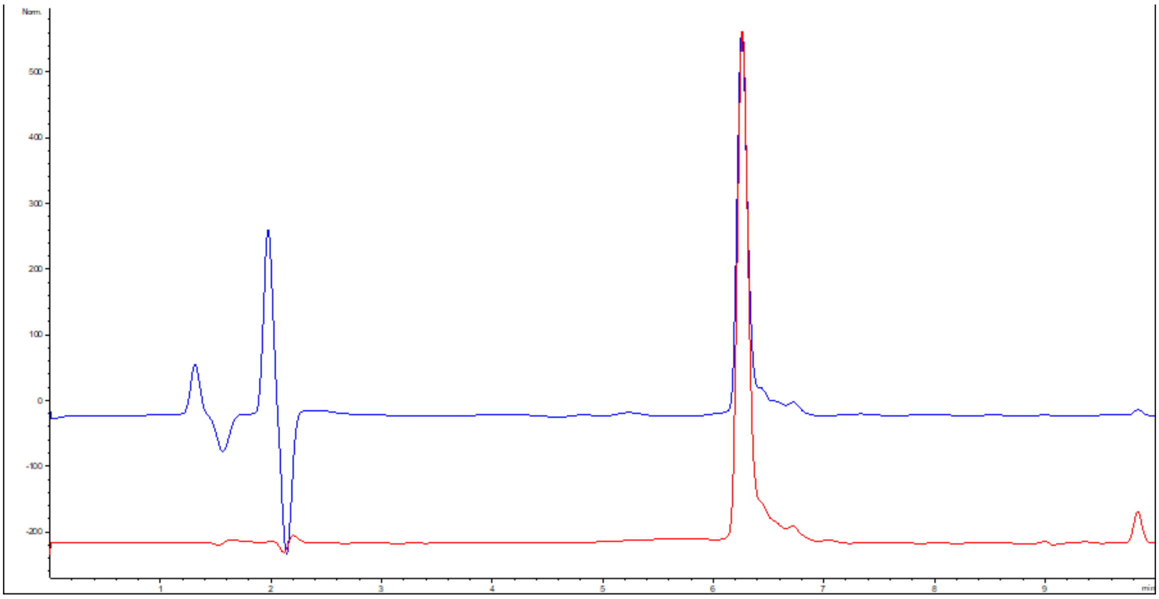

Figure S11. M4 HPLC trace, showing A215 in red and A280 in blue (Y-axis shown for A215, A280 trace autoscaled to fit).

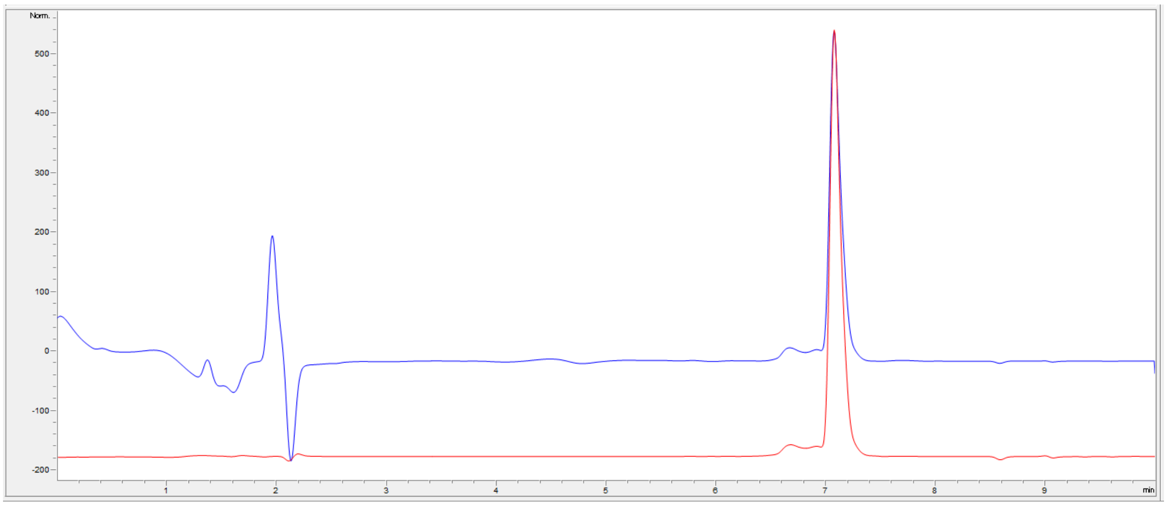

Figure S12. M5 HPLC trace, showing A215 in red and A280 in blue (Y-axis shown for A215, A280 trace autoscaled to fit).

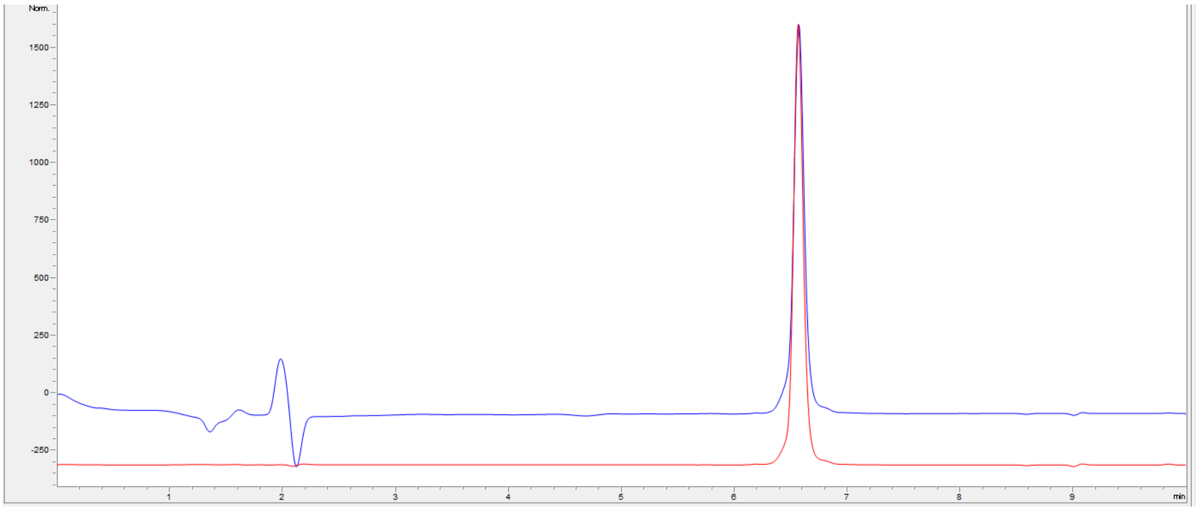

Figure S13. M6 HPLC trace, showing A215 in red and A280 in blue (Y-axis shown for A215, A280 trace autoscaled to fit).

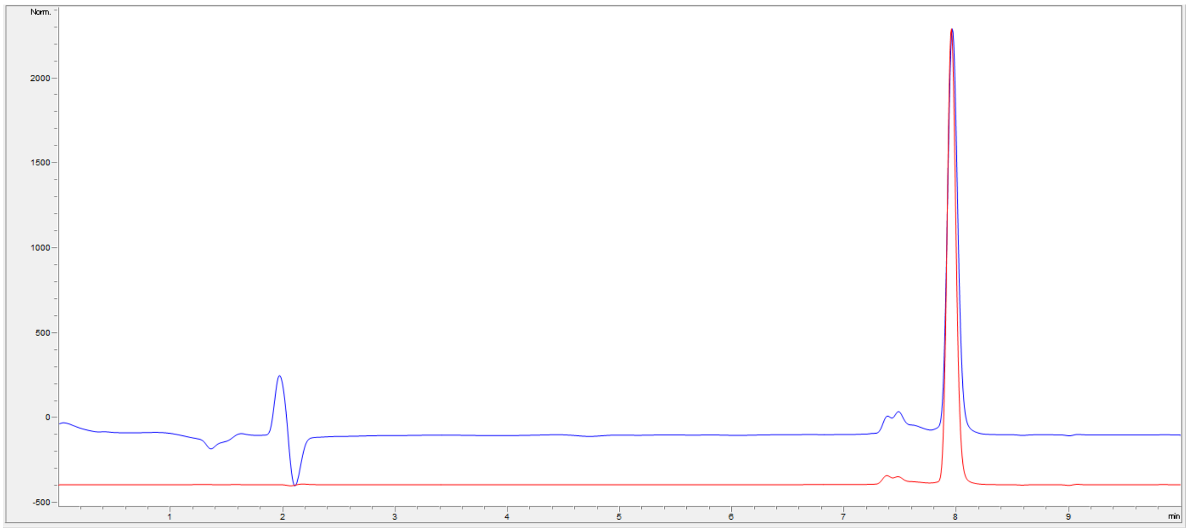

Figure S14. M7 HPLC trace, showing A215 in red and A280 in blue (Y-axis shown for A215, A280 trace autoscaled to fit).

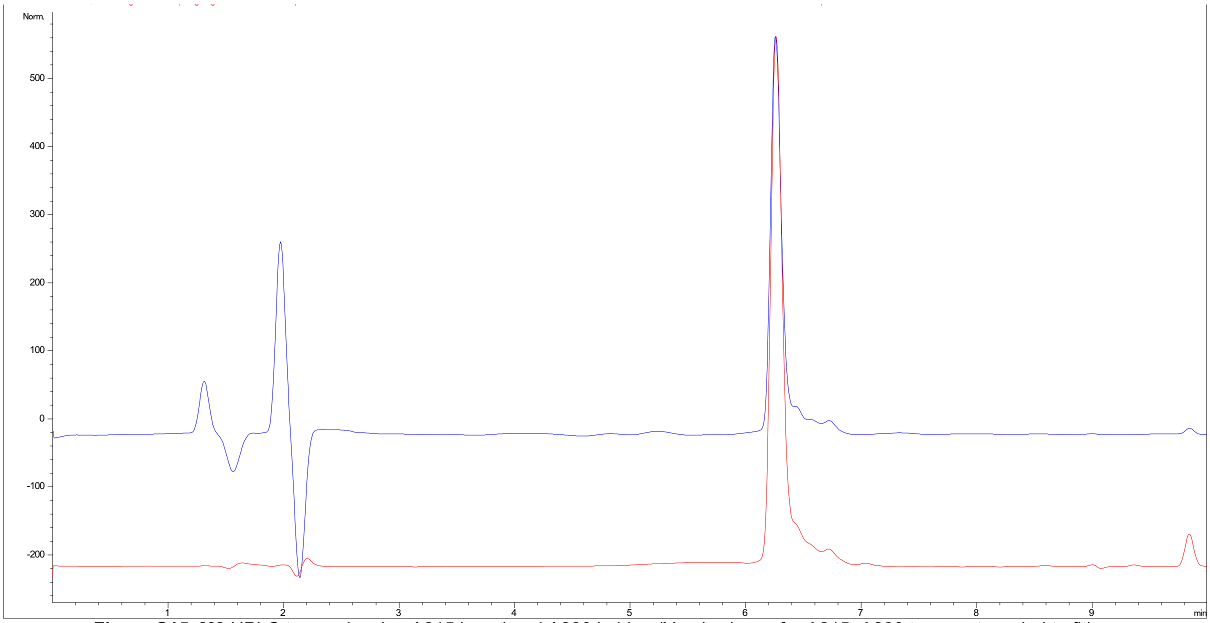

Figure S15. M8 HPLC trace, showing A215 in red and A280 in blue (Y-axis shown for A215, A280 trace autoscaled to fit).

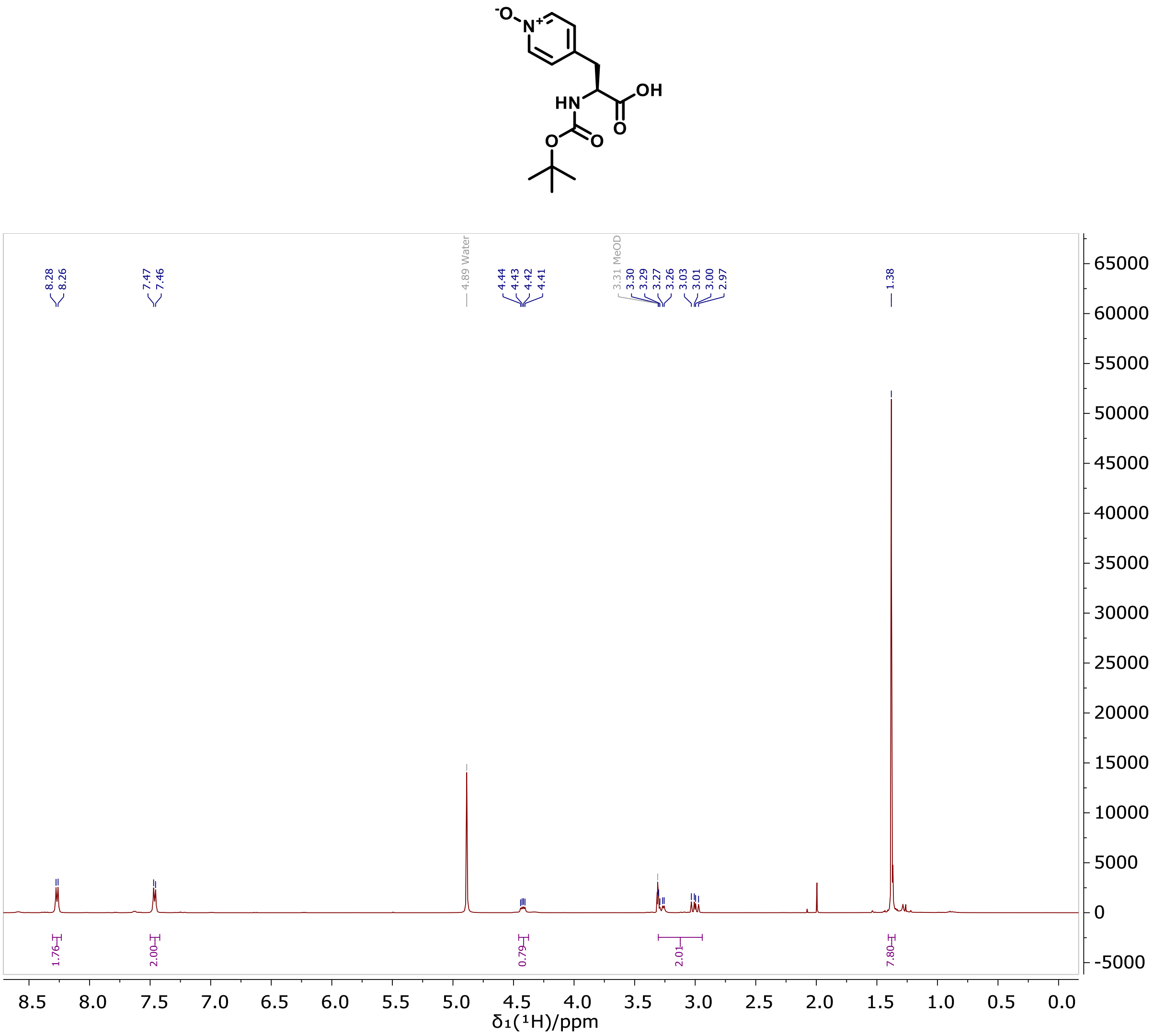

Figure S16. ^{1}H NMR of (*S*)-4-(2-((tert-butoxycarbonyl)amino)-2-carboxyethyl)pyridine 1-oxide.

.

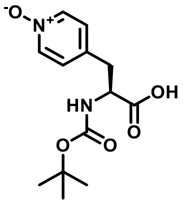

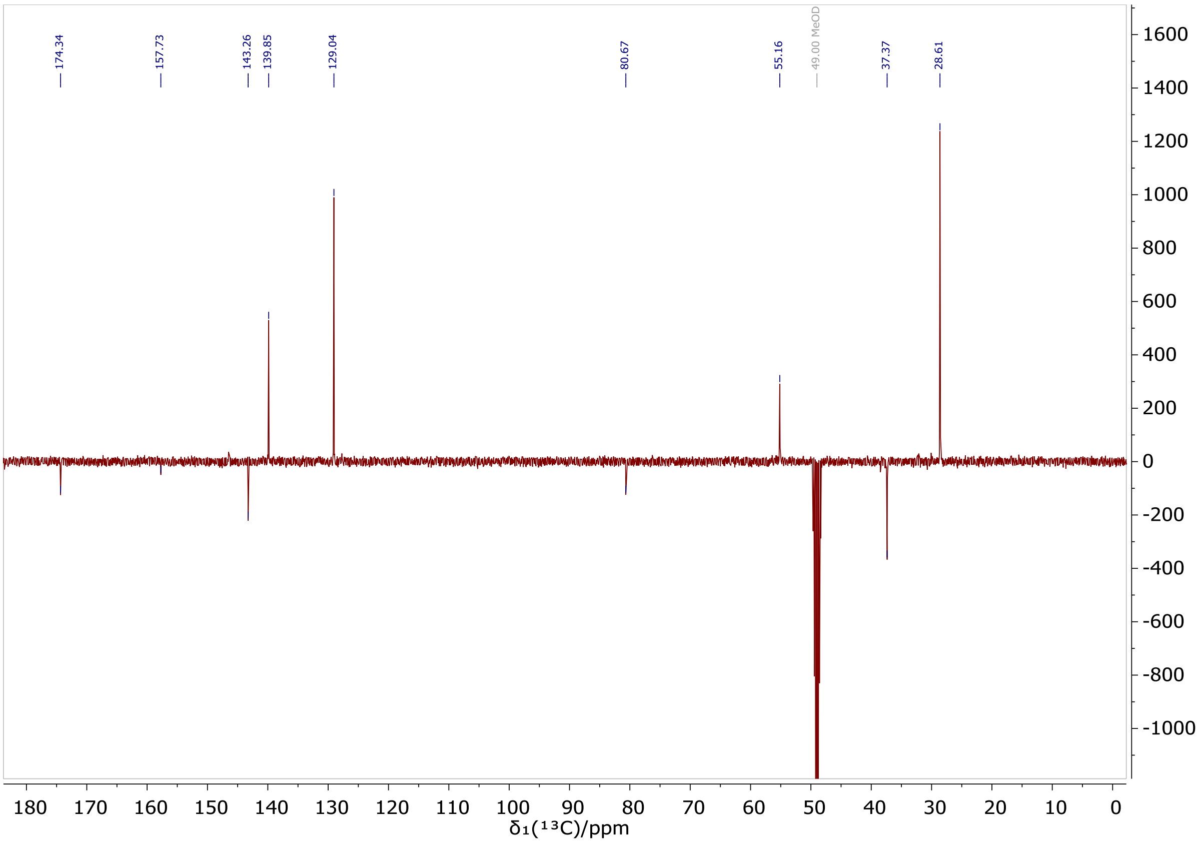

Figure S17. ^{13}C NMR of (*S*)-4-(2-((tert-butoxycarbonyl)amino)-2-carboxyethyl)pyridine 1-oxide.

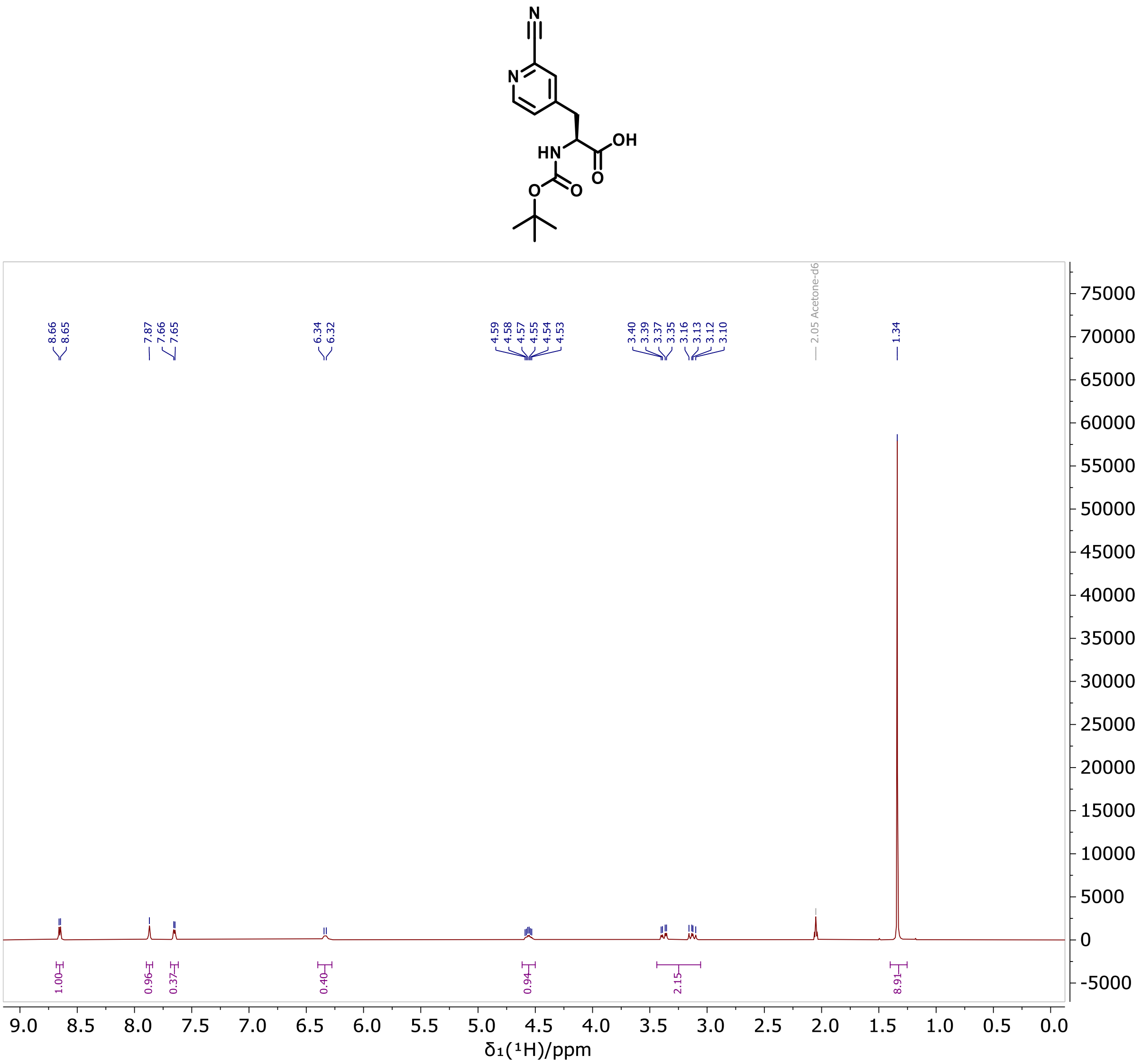

Figure S18. ^{1}H NMR of (*S*)-2-((*tert*-butoxycarbonyl)amino)-3-(2-cyanopyridin-4-yl)propanoic acid.

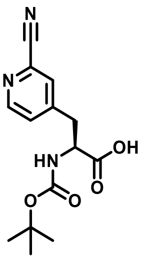

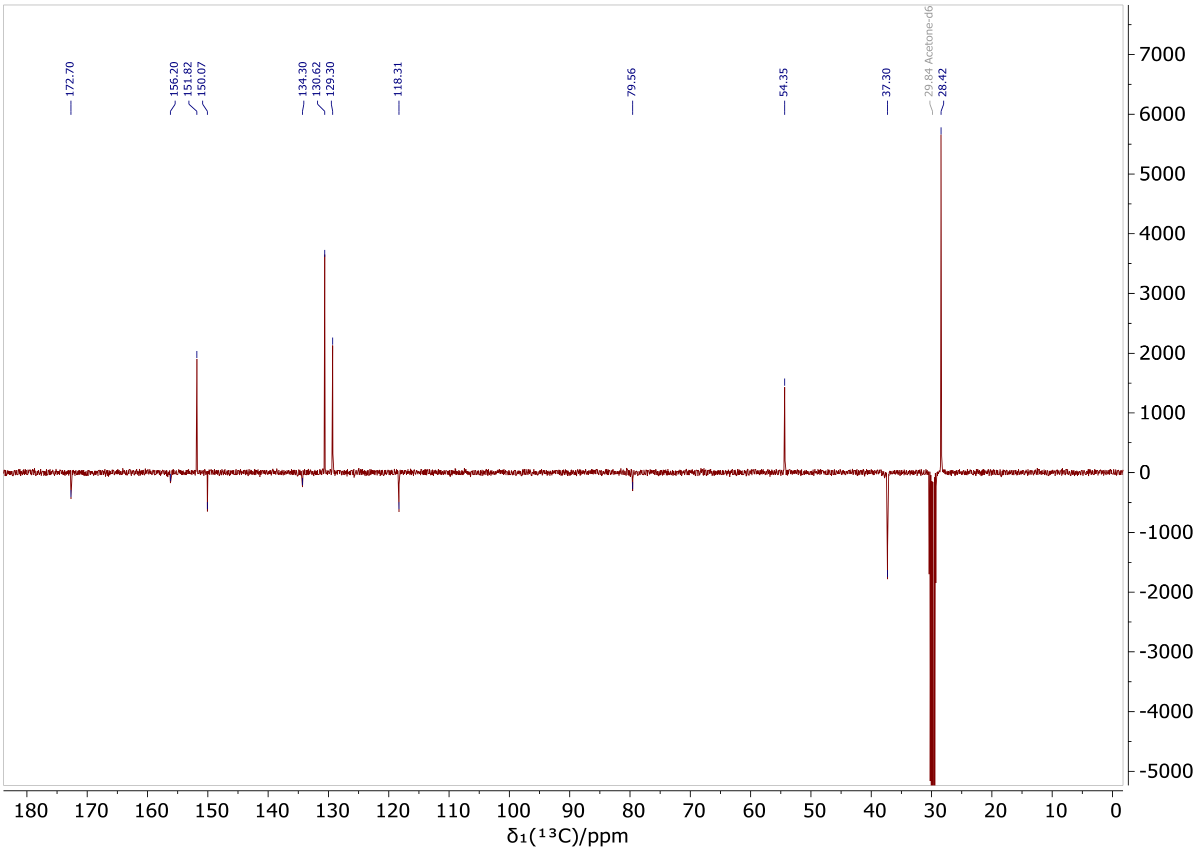

Figure S19. ^{13}C NMR of (*S*)-2-((*tert*-butoxycarbonyl)amino)-3-(2-cyanopyridin-4-yl)propanoic acid.

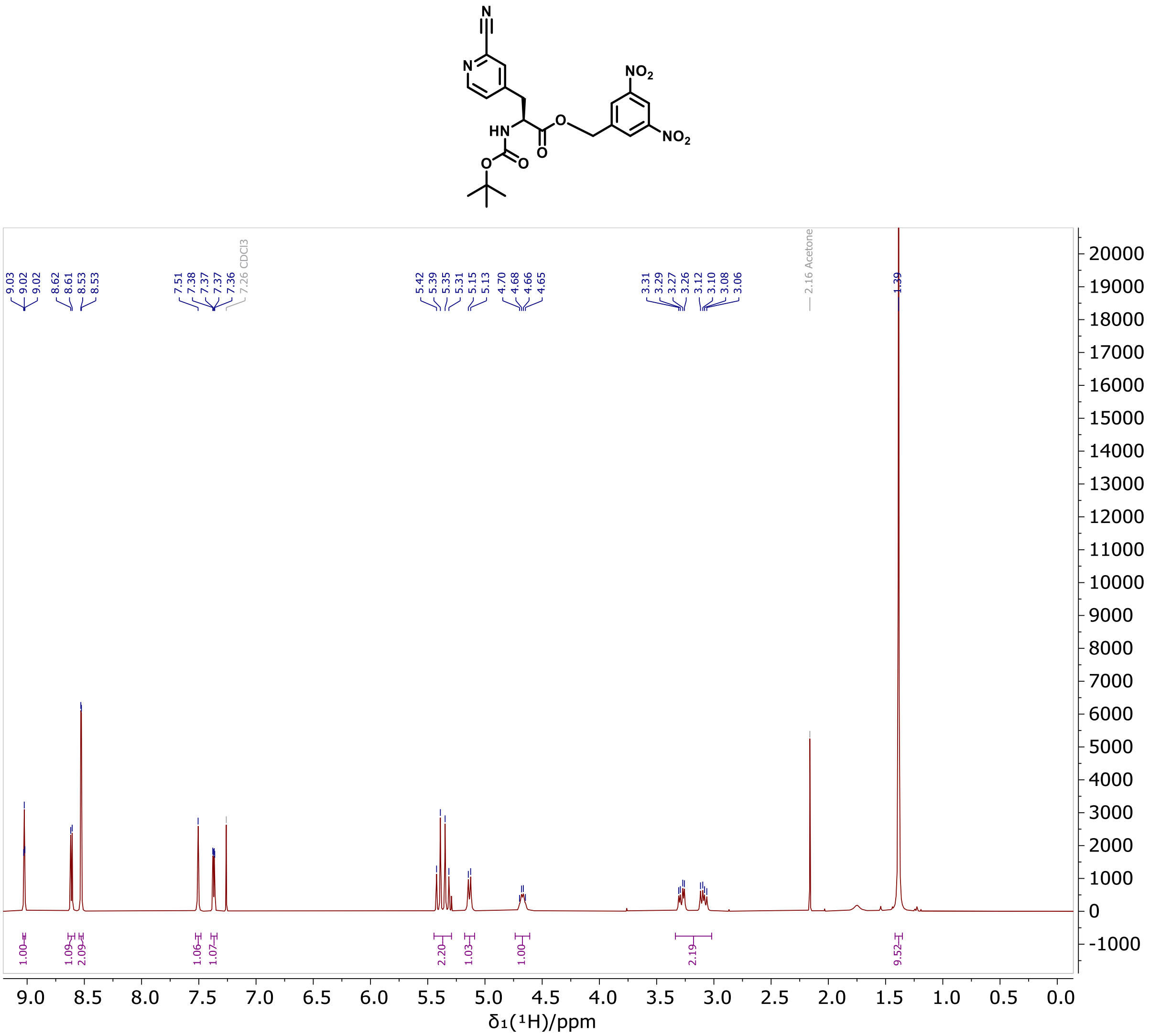

Figure S20. ^{1}H NMR of 3,5-dinitrobenzyl (S)-2-((*tert*-butoxycarbonyl)amino)-3-(2-cyanopyridin-4-yl)propanoate.

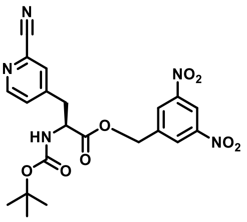

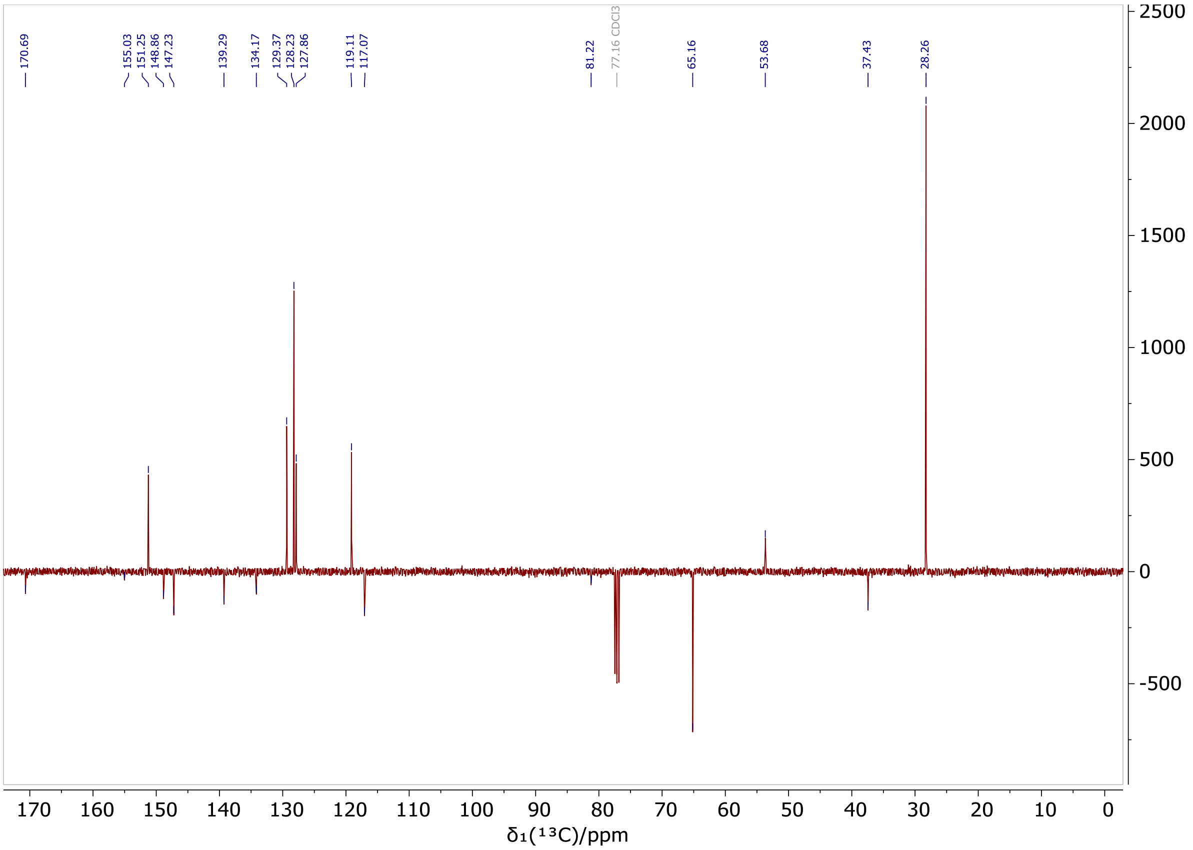

Figure S21. ^{13}C NMR of 3,5-dinitrobenzyl (*S*)-2-((*tert*-butoxycarbonyl)amino)-3-(2-cyanopyridin-4-yl)propanoate.

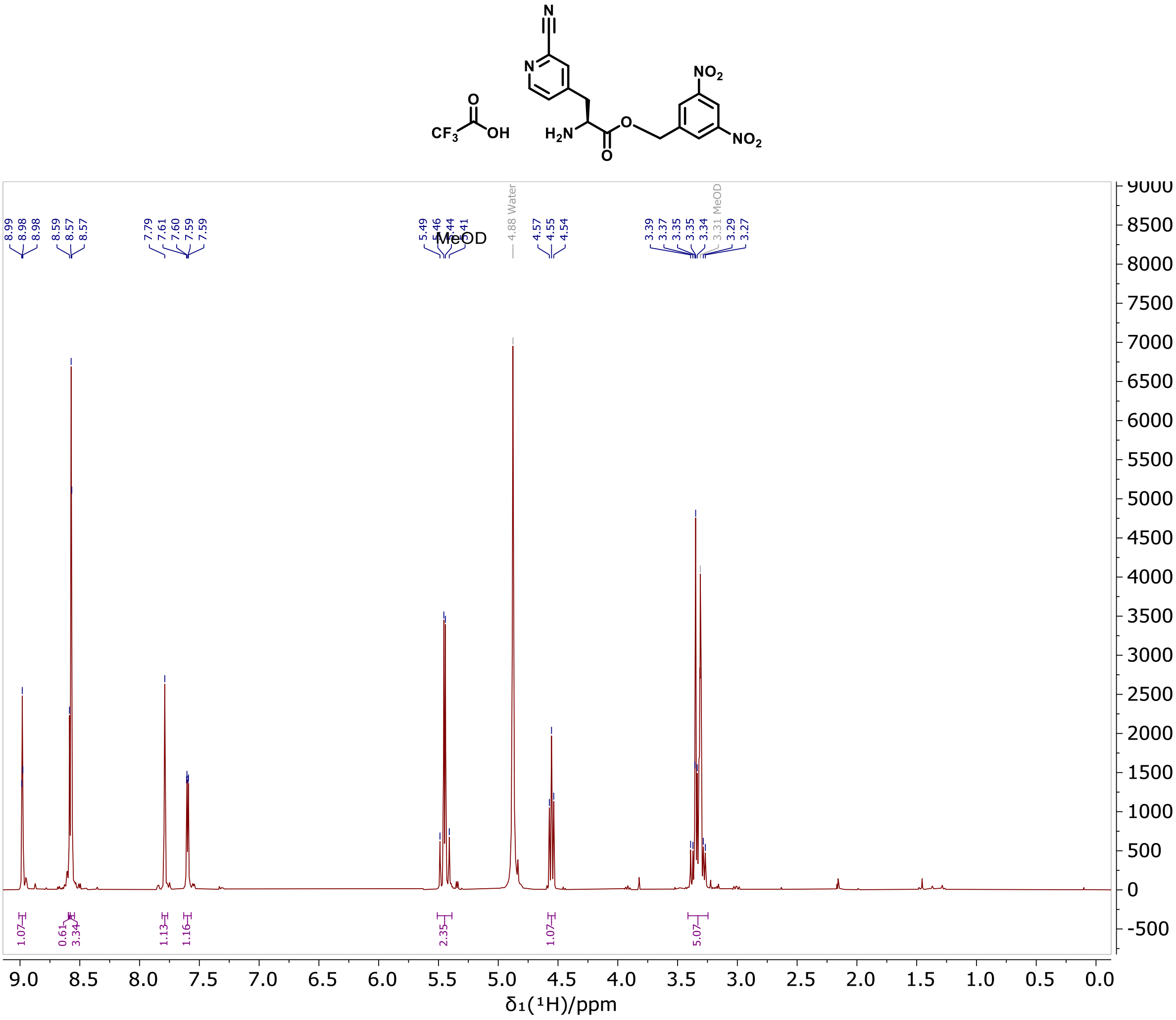

Figure S22. ^{1}H NMR of 3,5-dinitrobenzyl (*S*)-2-amino-3-(2-cyanopyridin-4-yl)propanoate.trifluoroacetate.

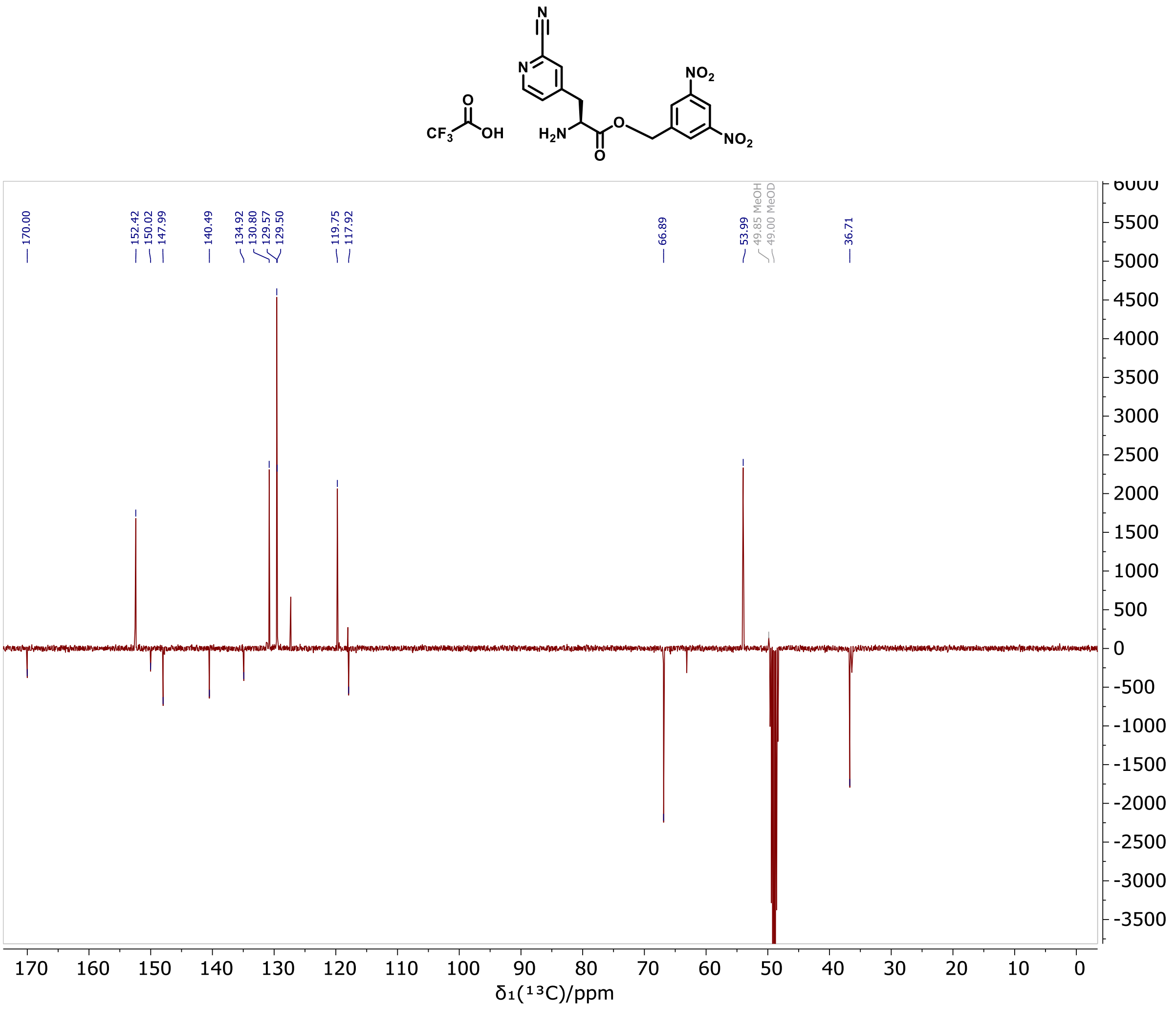

Figure S23. ^{13}C NMR of 3,5-dinitrobenzyl (*S*)-2-amino-3-(2-cyanopyridin-4-yl)propanoate.trifluoroacetate.

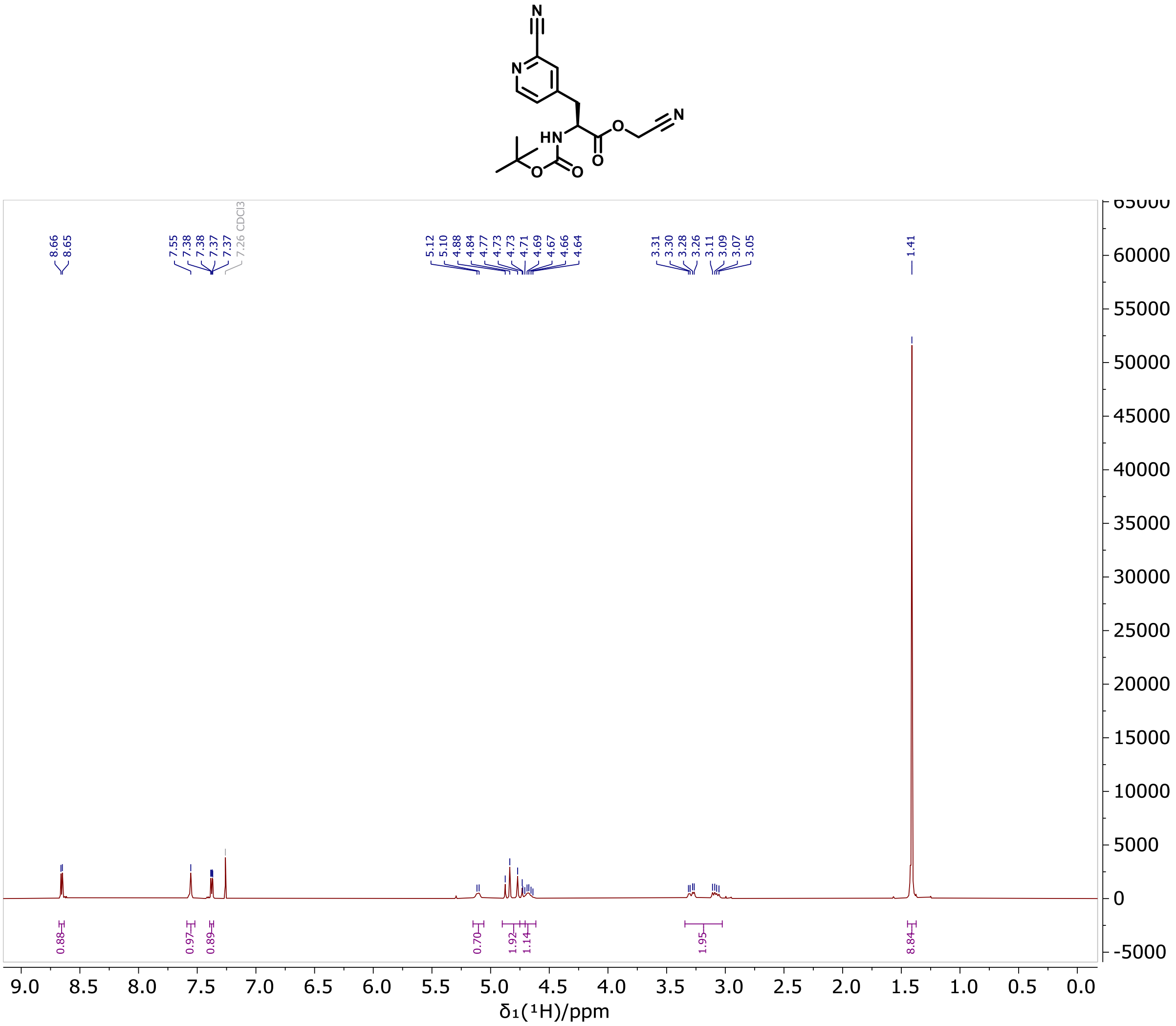

Figure S24. ¹H NMR of cyanomethyl (*S*)-2-((*tert*-butoxycarbonyl)amino)-3-(2-cyanopyridin-4-yl)propanoate.

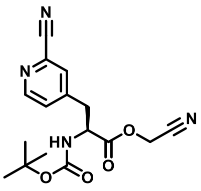

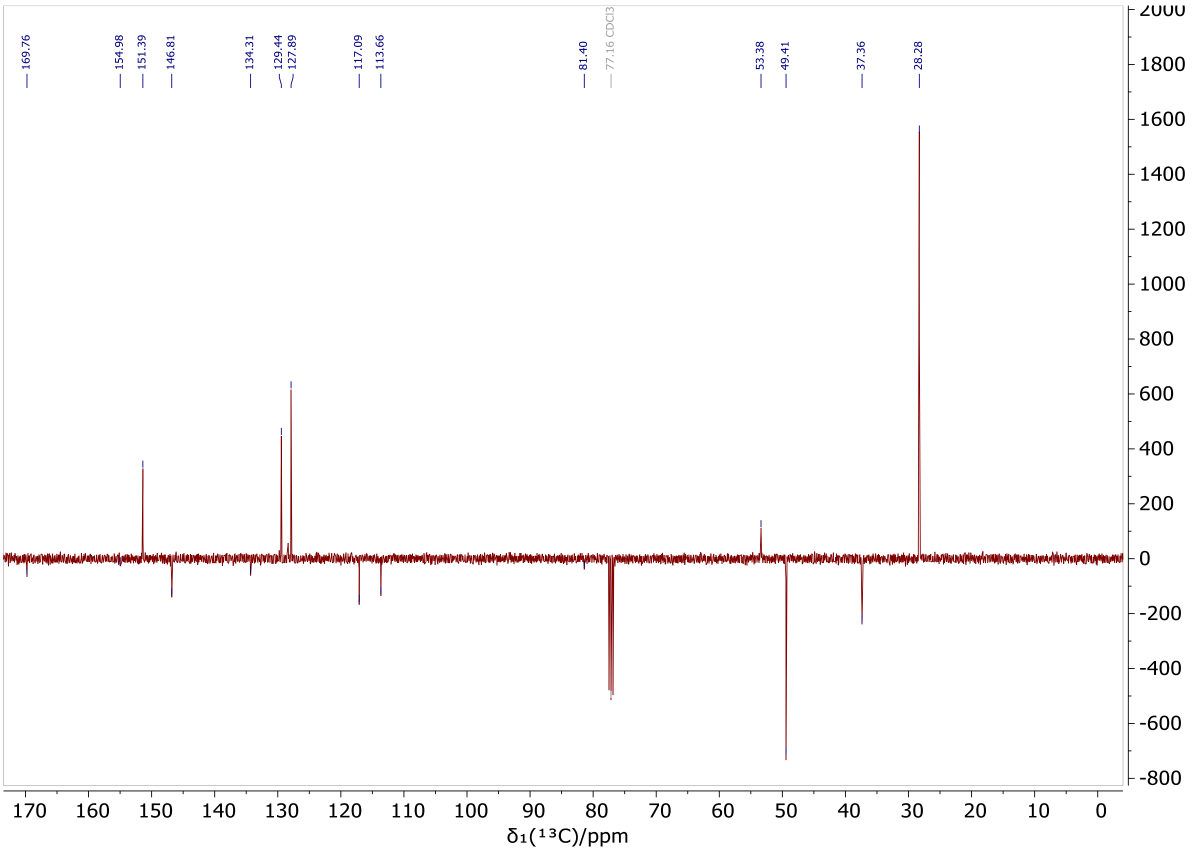

Figure S25. ^{13}C NMR of cyanomethyl (*S*)-2-((*tert*-butoxycarbonyl)amino)-3-(2-cyanopyridin-4-yl)propanoate.

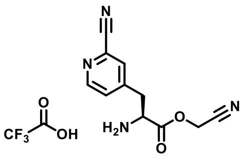

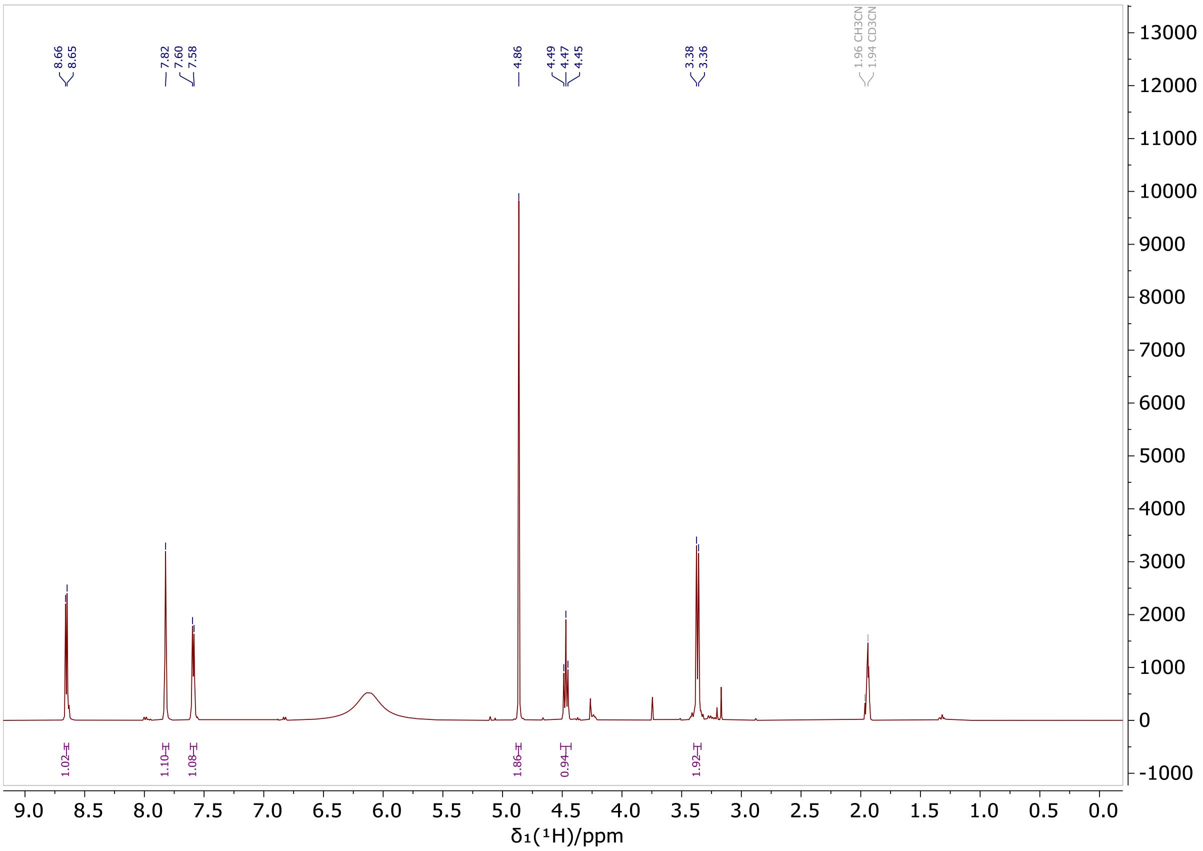

Figure S26. ^{1}H NMR of cyanomethyl (*S*)-2-amino-3-(2-cyanopyridin-4-yl)propanoate.trifluoroacetate.

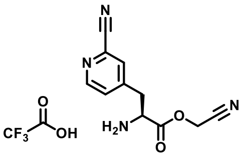

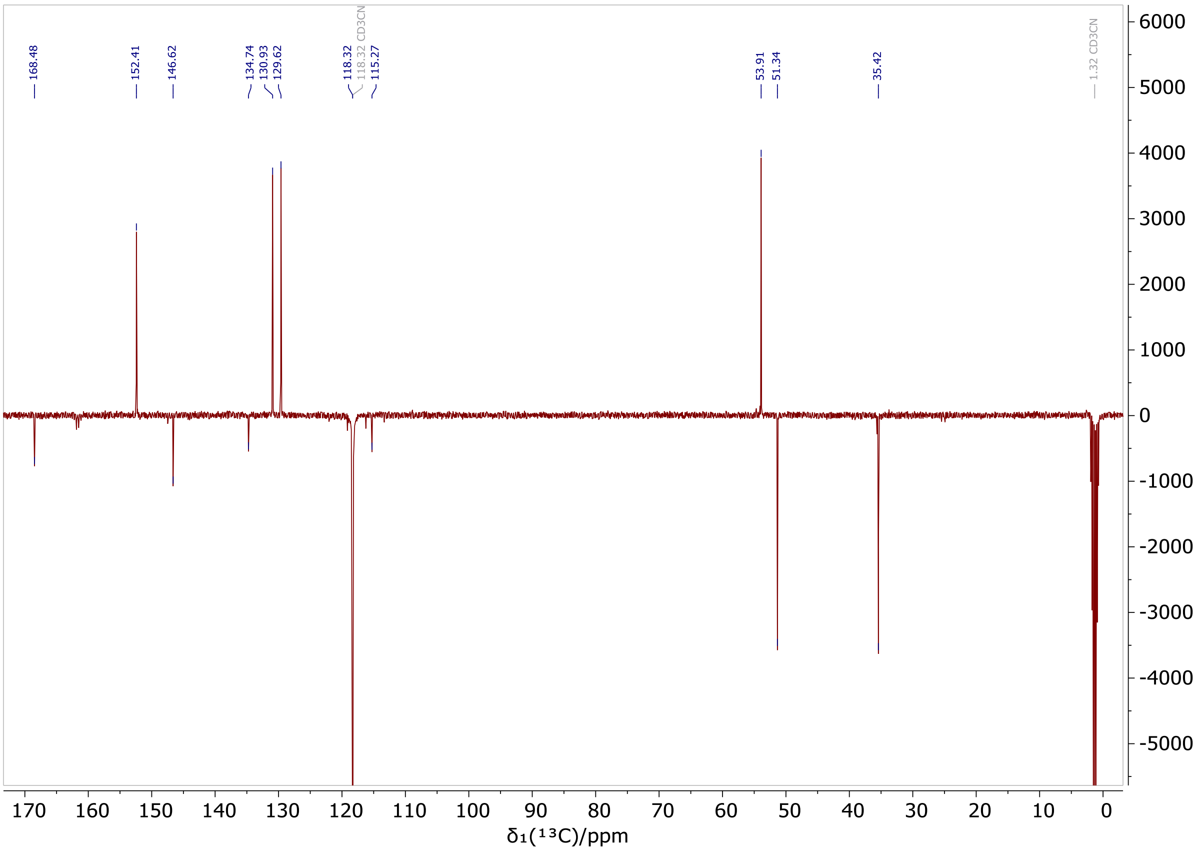

Figure S27. ^{13}C NMR of cyanomethyl (*S*)-2-amino-3-(2-cyanopyridin-4-yl)propanoate.trifluoroacetate.

References

1 Y. Goto, T. Katoh and H. Suga, Flexizymes for genetic code reprogramming., *Nat. Protoc.*, 2011, **6**, 779–790.

2 T. Fujino, T. Kondo, H. Suga and H. Murakami, Exploring of minimal RNA substrate of flexizymes, *ChemBioChem*, 2019, **20**, 1959–1965.

3 Z. Cui, Y. Wu, S. Mureev and K. Alexandrov, Oligonucleotide-mediated tRNA sequestration enables one-pot sense codon reassignment in vitro, *Nucleic Acids Res.*, 2018, **46**, 6387–6400.

4 M. N. Pascha, V. Thijssen, J. E. Egido, M. W. Linthorst, J. H. Van Lanen, D. A. A. Van Dongen, A. J. P. P. Hopstaken, F. J. M. Van Kuppeveld, J. Snijder, C. A. M. De Haan, S. A. K. K. Jongkees, J. H. Van Lanen, D. A. A. Van Dongen, A. J. P. P. Hopstaken, F. J. M. Van Kuppeveld, J. Snijder, C. A. M. De Haan and S. A. K. K. Jongkees, Inhibition of H1 and H5 Influenza A Virus Entry by Diverse Macrocyclic Peptides Targeting the Hemagglutinin Stem Region, *ACS Chem. Biol.*, 2022, **17**, 2425–2436.

5 T. Ishizawa, T. Kawakami, P. C. Reid and H. Murakami, TRAP display: A high-speed selection method for the generation of functional polypeptides, *J. Am. Chem. Soc.*, 2013, **135**, 5433–5440.

6 V. Thijssen, D. L. Hurdiss, O. J. Debski-Antoniak, M. A. Spence, C. Franck, A. Norman, A. Aggarwal, N. J. Mokiem, D. A. A. van Dongen, S. W. Vermeir, M. Liu, W. Li, M. Chatziandreou, T. Donselaar, W. Du, I. Drulyte, B. J. Bosch, J. Snijder, S. G. Turville, R. J. Payne, C. J. Jackson, F. J. M. van Kuppeveld and S. A. K. Jongkees, A broad-spectrum macrocyclic peptide inhibitor of the SARS-CoV-2 spike protein, *Proc. Natl. Acad. Sci. U. S. A., 2023, e2303292120.*

7 C. Nitsche, H. Onagi, J.-P. Quek, G. Otting, D. Luo and T. Huber, Biocompatible Macrocyclization between Cysteine and 2-Cyanopyridine Generates Stable Peptide Inhibitors, *Org. Lett.*, 2019, **21**, acs.orglett.9b01545.

8 J. Wang, W. Wang, P. A. Kollman and D. A. Case, Automatic atom type and bond type perception in molecular mechanical calculations, *J. Mol. Graph. Model.*, 2006, **25**, 247–260.

9 J. Yang and Y. Zhang, I-TASSER server: New development for protein structure and function predictions, *Nucleic Acids Res.*, 2015, **43**, W174–W181.

10 D. A. Case, H. M. Aktulga, K. Belfon, I. Y. Ben-Shalom, J. T. Berryman, S. R. Brozell, D. S. Cerutti, I. T.E. Cheatham, G. A. Cisneros, V. W. D. Cruzeiro, T. A. Darden, N. Forouzesh, G. Giambaşu, T. Giese, M. K. Gilson, H. Gohlke, A. W. Goetz, J. Harris, S. Izadi, S. A. Izmailov, K. Kasavajhala, M. C. Kaymak, E. King, A. Kovalenko, T. Kurtzman, T. S. Lee, P. Li, C. Lin, J. Liu, T. Luchko, R. Luo, M. Machado, V. Man, M. Manathunga, K. M. Merz, Y. Miao, O. Mikhailovskii, G. Monard, H. Nguyen, K. A. O’Hearn, A. Onufriev, F. Pan, S. Pantano, R. Qi, A. Rahnamoun, D. R. Roe, A. Roitberg, C. Sagui, S. Schott-Verdugo, A. Shajan, J. Shen, C. L. Simmerling, N. R. Skrynnikov, J. Smith, J. Swails, R. C. Walker, J. Wang, J. Wang, H. Wei, X. Wu, Y. Wu, Y. Xiong, Y. Xue, D. M. York, S. Zhao, Q. Zhu and P. A. Kollman, Amber 2023, University of California, San Francisco.
